# Sequence variation in *Plasmodium falciparum* merozoite surface protein-2 is associated with virulence causing severe and cerebral malaria

**DOI:** 10.1371/journal.pone.0190418

**Published:** 2018-01-17

**Authors:** Suwanna Chaorattanakawee, Pornlada Nuchnoi, Hathairad Hananantachai, Uranan Tumkosit, David Saunders, Izumi Naka, Jun Ohashi, Jintana Patarapotikul

**Affiliations:** 1 Department of Medical Technology, Faculty of Allied Health Sciences, Thammasat University, Rangsit, Patumthani, Thailand; 2 Department of Parasitology and Entomology, Faculty of Public Health, Mahidol University, Bangkok, Thailand; 3 Department of Clinical Microscopy, Faculty of Medical Technology, Mahidol University, Nakhon pathum, Thailand; 4 Department of Social and Environmental Medicine, Faculty of Tropical Medicine, Mahidol University, Bangkok, Thailand; 5 Department of Microbiology and Immunology, Faculty of Tropical Medicine, Mahidol University, Bangkok, Thailand; 6 US Army Medical Materiel Development Activity, Fort Detrick, Maryland, United States of America; 7 Department of Biological Sciences, Graduate School of Science, The University of Tokyo, Tokyo, Japan; Liverpool School of Tropical Medicine, UNITED KINGDOM

## Abstract

Parasite virulence, an important factor contributing to the severity of *Plasmodium falciparum* infection, varies among *P*. *falciparum* strains. Relatively little is known regarding markers of virulence capable of identifying strains responsible for severe malaria. We investigated the effects of genetic variations in the *P*.*f*. merozoite surface protein 2 gene (*msp*2) on virulence, as it was previously postulated as a factor. We analyzed 300 *msp2* sequences of single *P*. *falciparum* clone infection from patients with uncomplicated disease as well as those admitted for severe malaria with and without cerebral disease. The association of *msp2* variations with disease severity was examined. We found that the N allele at codon 8 of Block 2 in the FC27-like *msp2* gene was significantly associated with severe disease without cerebral complications (odds ratio = 2.73, *P* = 0.039), while the K allele at codon 17 of Block 4 in the 3D7-like *msp2* gene was associated with cerebral malaria (odds ratio = 3.52, *P* = 0.024). The data suggests possible roles for the associated alleles on parasite invasion processes and immune-mediated pathogenicity. Multiplicity of infection was found to associate with severe disease without cerebral complications, but not cerebral malaria. Variations in the *msp2*-FC27-block 2-8N and 3D7-block 4-17K allele appear to be parasite virulence markers, and may be useful in determining the likelihood for severe and cerebral malaria. Their interactions with potential host factors for severe diseases should also be explored.

## Introduction

The clinical presentation of malaria caused by *P*. *falciparum* ranges from asymptomatic infection to moderate acute febrile illness to severe complicated disease with organ failure, including life-threatening cerebral malaria. Although factors contributing to this wide spectrum of severity have not been well characterized to date, parasite virulence is believed to be an important contributor [1]. Multiple field studies have tried to characterize virulent strains of *P*. *falciparum* using genetic polymorphisms as markers [2–8]. Although evidence of differences in virulence among *P*. *falciparum* strains have accumulated, the virulent strains have not yet been characterized in sufficient detail to identify suitable virulence markers.

Merozoite surface protein 2 gene *(msp2)* has been shown to be a useful marker for strain differentiation [9, 10]. *Msp2* is involved in RBC invasion, as anti- *msp2* antibodies have been shown to inhibit merozoite invasion and parasite growth [11, 12]. Synthetic *msp2* peptides bind with high affinity to RBCs, and can also inhibit parasite invasion [13]. Moreover, *msp2* has been implicated as a target of naturally acquired clinical immunity to malaria [14–18], and used as a candidate malaria vaccine antigen [12, 19, 20]. *Msp2* is exceptionally interesting as a candidate marker for parasite virulence given its pathogenicity and genetic diversity, with a high degree of both length and sequence polymorphism [21]. It is composed of five domains including conserved N- and C-terminal domains (block 1 and block 5), two non-repetitive variable domains (block 2 and block 4) and a central repetitive domain (block 3). Sequences in block 2 and block 4 are dimorphic and used as the basis to divide *msp2* alleles into two distinct families—FC27 and IC-1/3D7. Repeated sequences in block 3 vary in number and sequence of repeat units [21–23].

Although *msp2* is highly polymorphic, most studies to date have used only *msp2* allelic dimorphism and size variation as markers for parasite virulence genotyping based on PCR followed by a conventional gel electrophoresis or high-resolution capillary electrophoresis to analyze fragment sizes. As yet, no conclusive relationships with virulence have been observed. Previous studies have shown mixed results with both the FC27 and 3D7 families variously described as virulent strains. Virulence of particular families also varied between communities [5, 6, 24, 25], and several studies could not find any association between particular alleles and disease severity [2, 3, 26, 27]. These inconsistent results need to be verified, although it may be partly explained by variability in genotyping and interpretation methods of those studies, as well as parasite heterogeneity among the populations studied. Sequence analysis may detect variation in dimorphic and repetitive regions more sensitively, possibly providing useful information to characterize parasite virulence. In this study, parasite *msp2* sequences from Thai malaria patients with mild to severe cerebral clinical disease were analyzed to evaluate associations with malaria severity.

## Materials and methods

### Patients

A total of 480 *P*. *falciparum*-infected blood samples were analyzed in this study. Samples were obtained from patients living in northwest Thailand near the Myanmar border. *P*. *falciparum* infection was diagnosed by microscopic examination of giemsa-stained thick and thin blood films. Patients were classified clinically into 3 groups: mild (n = 204), non-cerebral severe (n = 166), and cerebral (n = 110) malaria. Cerebral malaria was characterized by unrousable coma with positive asexual *P*. *falciparum* forms and exclusion of other causes of coma. Severe, non-cerebral malaria was characterized by one of the following symptoms: high parasitemia (>100,000 parasites/μl), hypoglycemia (glucose < 2.2 mmole/liter), severe anemia (haematocrit < 20% or haemoglobin < 7.0 g/dl), and increased serum creatinine levels (> 3.0 mg/dl). Non-cerebral severe malaria is referred to as ‘severe malaria’ for the remainder of the paper. Mild malaria was characterized by a positive blood smear, fever without other identified cause of infection, and the absence of manifestations of severe or cerebral malaria as described above [28]. Patients underwent clinically appropriate treatment based on presenting clinical features at the hospital for Tropical Disease, Faculty of Tropical Medicine, Mahidol University, Bangkok, Thailand. All subjects were ≥ 13 years old with a mean age of 25.5, 23.9, and 28.6 years for mild, severe, and cerebral malaria, respectively. Average parasite density for mild, severe, and cerebral malaria were 28,577, 165,166, and 111,686 parasites/μl, respectively. This study was approved by the institutional review boards of Thammasat University, Thailand. Prior to enrollment, written informed consent was obtained from all participants or their parents or guardians for those under 18 years of age.

### Blood collection and DNA preparation

Blood samples were collected at the time of diagnosis and prior to treatment in EDTA tubes. Genomic DNA was purified from whole blood using a QIAamp miniblood kit (QIAGEN, Hilden, Germany) according to the manufacturer’s instructions.

### *Msp2* amplification and sequencing

Nested PCR amplification of the *P*. *falciparum msp2* gene was performed in all 480 samples using 2 pairs of primers, using previously described primer sequences and PCR conditions [29]. PCR products were analyzed on 6% polyacrylamide gel with a standard molecular weight (100bp DNA ladder, Takara, Japan). Multiple infections with two or more parasite clones were defined as >1 band of amplified PCR product observed. Since in pre-analysis of this dataset, multiplicity of infection was found to be a confounding factor for malaria severity, only samples with single clone infection were analyzed for *msp2* sequence. Direct sequencing of PCR products was performed on both strands of DNA using the BigDye® Terminator V3.1 Cycle Sequencing Kit (Applied Biosystems, Foster City. CA, USA).

### Sequences and polymorphisms analysis

Variable *msp2* sequence domains including block 2, 3, and 4 were analyzed for polymorphisms. Based on sequence similarity to *msp2* sequences of the K1 (FC27-liked) and 3D7 strains (GenBank accession numbers: M59766.1; PFB0300c), *msp2* sequences obtained here were grouped into 2 allelic families—FC27 and 3D7. Since block 2, 3, and 4 of *msp2* were absolutely different among these 2 families, separate alignment of each family was performed to analyze for polymorphisms. Nucleotide alignments were performed separately for each block using Bioedit software [30]. Blocks and their borders were defined as described by Ferreira et al. [21]. For repeat region in block 3, nucleotide / amino acid sequences of repeat units were aligned and variations of both copy number and sequences of repeat units were analyzed. These polymorphic sequences were submitted to the GenBank database (accession numbers JX885898-JX885980).

### Statistical analysis

To determine the possible association of *msp2* with malaria severity, allele and haplotype frequencies of *msp2* were compared between the mild and severe, mild and cerebral, and severe and cerebral malaria groups using the χ^2^ test or Fisher’s exact test. In this study, only alleles / haplotypes with frequency ≥ 10% were included in statistical analyses. Association analysis was conducted separately for the two allelic families. To study the association of *msp2* with parasitaemia, parasitaemia levels at presentation were compared between patients carrying different alleles and haplotypes of *msp2* using the Mann Whitney U test, and Kruskal-Wallis test, respectively. The analysis were performed by SPSS 18 for windows (SPSS, Inc., Chicago. IL). The extent of linkage disequilibrium (LD) between bi-allelic polymorphisms was evaluated by r^2^ values calculated using the Haploview software [31]. For all analysis, a *P* value of less than 0.05 was considered to be statistically significant.

## Results

### Multiplicity of *P*. *falciparum* infection associated with severe malaria

Of 480 blood samples, *msp2* amplification was successful in 471 samples (98.1%), generating 400–800 bp PCR products. Based on number of amplified *msp2* bands, multiple infections were found in 166 (35%) samples, including 124 double infection (26%), 30 triple infection (6%), 6 samples with 4 clones infection (1%), 4 samples with 5 clones infection (0.9%), and 2 samples with 6 clones infection (0.4%). Infection with multiple *P*. *falciparum* clones was associated with increased risk of non-cerebral severe malaria (χ^2^ for trend, *P* = 0.012), notably when infection with more than 2 clones (Table 1). Patients who were infected with more than 2 parasite clones had higher risk to severe malaria compared to those infected with 2 or 1 parasite clone with OR of 2.5, *P* = 0.008. Increase in multiplicity of infection (MOI) associated with high parasitemia, as median of 25,890, 22,740, 99,200, 191,930 parasite/μL for patients infected with 1, 2, 3, and >3 parasite clones, respectively, suggesting that the apparent association of MOI with severe malaria was due to hyperparasitaemia. There was no association between MOI and cerebral malaria. However, when MOI among severe and cerebral malaria patients were compared, multiple clones infection was more common in severe malaria (43.3%) than cerebral malaria (24.5%).

**Table 1:** Multiplicity of *P*. *falciparum* infection among mild, severe and cerebral malaria patients.

Multiplicity of infection	Mild (%) N = 178	Severe (%) N = 105	Cerebral (%) N = 154	Odds for severe (mild vs severe)	Odds for cerebral (mild vs cerebral)
1 clone	129 (65.5)	93 (56.7)	83 (75.5)	0.72	0.64
2 clones	55 (27.9)	46 (28.1)	23 (20.9)	0.84	0.42
3 clones	10 (5.1)	18 (11.0)	2 (1.8)	1.8	0.20
> 3 clones	3 (1.5)	7 (4.3)	2 (1.8)	2.3	0.67

### *Msp2* sequences

**Fig 1:**
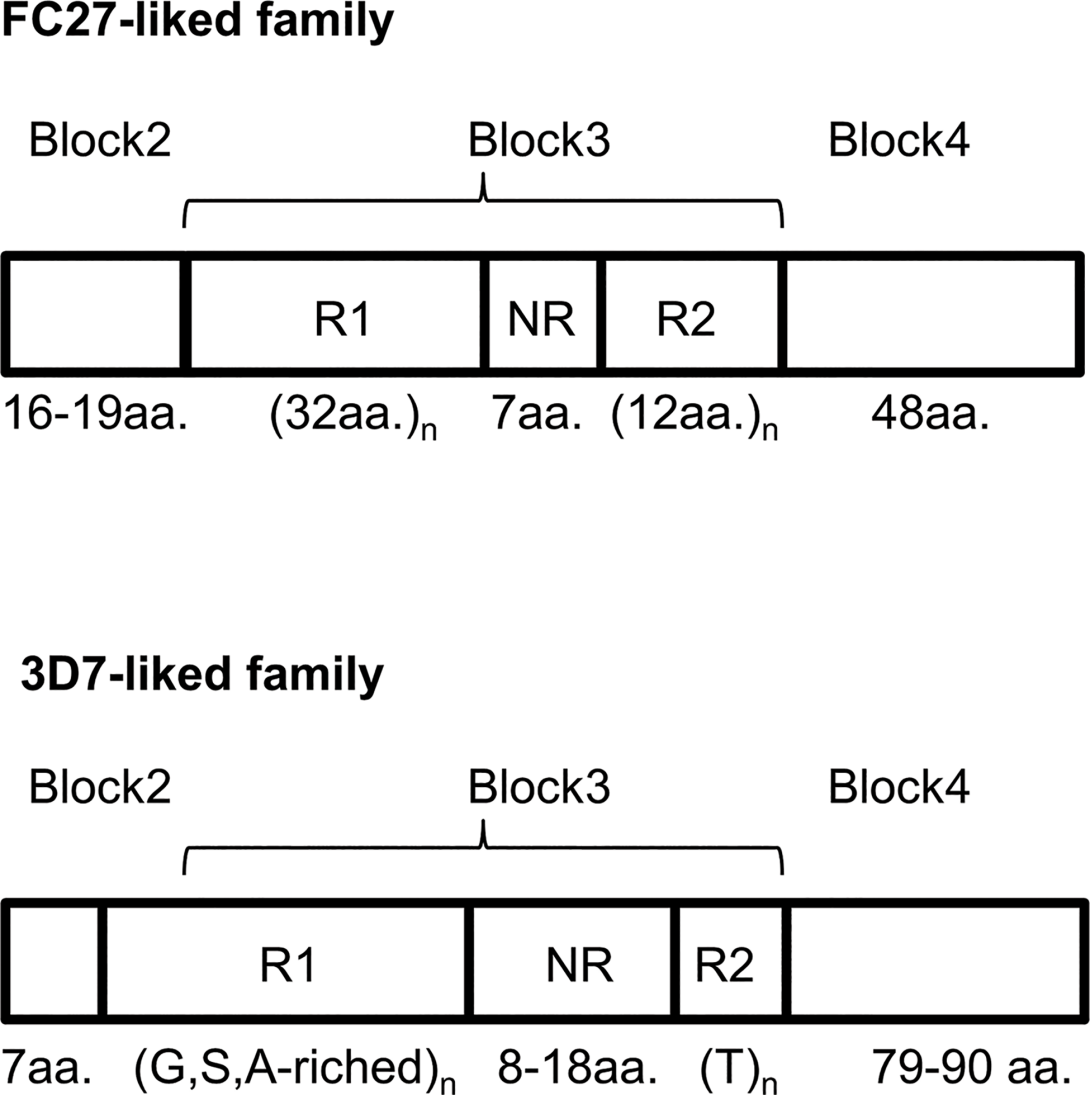
Structure of the variable region of the *Plasmodium falciparum msp2* gene showing block 2, 3, and 4. FC27 and 3D7-like structures were presented in the upper and lower panels, respectively. Amino acid (aa.) lengths of each block are indicated. Block 3 consist of 2 different repeat units (R1 and R2), which were separated by a non repeat region (NR). For 3D7-like sequences, R1 had Glycine (G), Serine (S), and Alanine (A) enriched sequences, while R2 featured Threonine (T) repeats.

Since multiplicity of infection was found as a confounder for malaria severity, only single clone infections were analyzed for *msp2* sequence and association with disease severity. Of 305 samples with single clone infection, 300 samples were available for *msp2* sequences analysis. DNA sequencing was successful in 277 samples (92.3%). Since the primers bind to the conserved domains (block 1 and block 5), only sequences of *msp2* variable domains, including block 2, 3 and 4 were obtained (Fig 1). Block 2 and block 4 were non-repetitive, dimorphic sequences, dividing *msp2* into two families—FC27 and 3D7-like. For block 3, two repetitive regions were found, designated as repeat region 1 (R1) and repeat region 2 (R2) and these two regions were separated by a non-repetitive region designated as the non-repeat region (NR) (Fig 1). Of 277 *msp2* sequences from single-clone infections, 114 (41.2%) belonged to the FC27 family, while 163 (58.8%) belonged to the 3D7-like family (Table 2). Sizes of FC27 and 3D7 variants ranged from 450–606 bp and 490–730, respectively (S1 Fig). Sequence analysis can define larger repertoire of distinct size variants (50 variants) of *msp2* (FC27 and 3D7) compared to simple gel electrophoresis (20 variants). Frequencies of sized variants based on both methods are shown in S1 Fig.

**Table 2:** Frequencies of FC27 and 3D7-like *msp2* in *P*. *falciparum* isolates from 277 malaria patients in Thailand with mild (M), severe (S) or cerebral (C) disease.

*Msp2*	Mild (M) N = 115	Severe (S) N = 84	Cerebral (C) N = 78	Total N = 277	M vs S *P*-value	M vs C *P*-value	S vs C *P*-value
family	(%)	(%)	(%)	(%)	Odds Ratio	Odds Ratio	Odds Ratio
FC27	54 (47.0)	28 (33.3)	32 (41.0)	114 (41.2)	*P* = 0.054	*P* = 0.416	*P* = 0.311
3D7	61 (53.0)	56 (66.7)	46 (59.0)	163 (58.8)	OR = 0.56	OR = 0.79	OR = 1.39

### *Msp2* alignment

**Fig 2:**
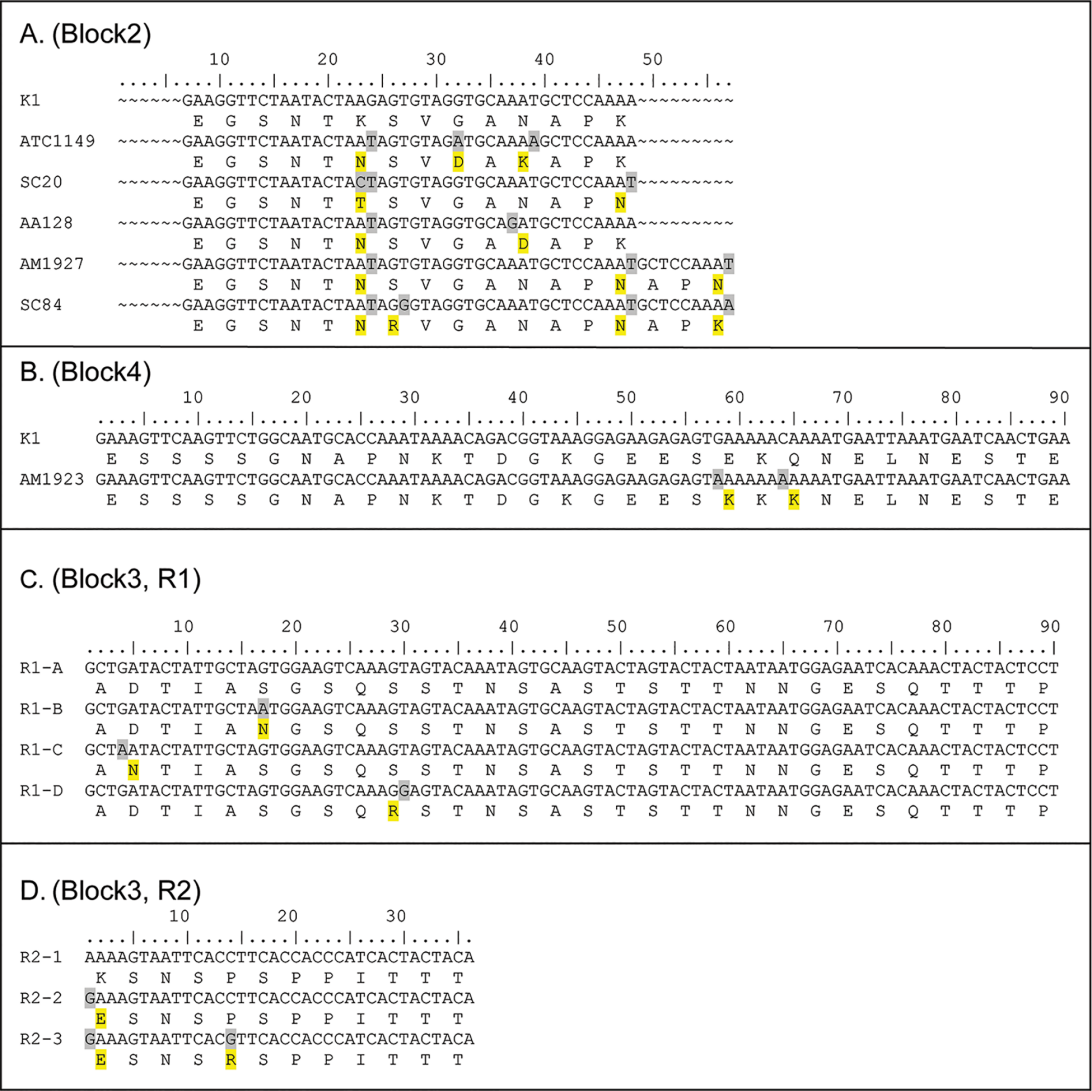
Sequence alignment of *Msp2* FC27 family of *Plasmodium falciparum* showing the polymorphisms in each block. Changes of nt. / aa. are shaded with gray / yellow. The *Msp2* sequence of K1 (FC27-liked) (GenBank accession number: M59766.1) was used as a reference for Block 2 and 4 alignment. (A) Block 2: the first 6 nt. residues of 57 (19 aa.) were not analyzed. An indel and 7 non-synonymous SNPs are shown. (B) Block 4: only the first 90 nt. residues of 144 (48 aa.) are presented. Two non-synonymous SNPs are shown. (C) Block 3, repeat region1 (R1): only the first 90 nt. residues of the 96 nt. repeat are presented, showing 4 repeat variants (R1-A, R1-B, R1-C, R1-D) with different positions of single non-synonymous SNPs. (D) Block 3, repeat region2 (R2): 36 nt. repeat showing 3 repeat variants (R2-1, R2-2, R2-3) with different non-synonymous SNPs.

Nucleotide alignment of 114 FC27-liked sequences demonstrated several polymorphisms. In block 2, an indel and 7 SNPs causing amino acids changes were found (Fig 2A, Table 3), while in block 4, two non-synonymous SNPs were found (Fig 2B, Table 3). In block 3, R1 contained one or three copies of the 96 nucleotide repeat (32 aa.), while R2 contained one to five copies of the 36 nucleotide repeat (12 aa.) generating 5 different combinations [(R1)_n_(R2)_n_] (Table 3). The non repeat region (NR) in this family contained conserved 21 nucleotides (7 aa.). Within the repeat of 96 nucleotides in R1 region, a single non-synonymous point mutations was found at position 17, 4 or 30, producing four variants [R1-A (no mutation), R1-B, R1-C, and R1-D respectively] (Fig 2C). Similarly for the R2 region, point mutations within the repeat of 36 nucleotides were found producing three variants; R2-1 (no mutation), R2-2 (single mutation at position 1), and R2-3 (double mutations at position 1 and 14) (Fig 2D). Considering both the copy number and the repeat variants, 13 allelic variants were found among the FC27-like *msp2* sequences (Table 3). Overall, the combination of block 2, 3, and 4 polymorphisms generated 21 distinct haplotypes among 114 FC27-like *msp2* sequences (Table 4) (GenBank accession numbers JX885898-JX885918).

**Table 3:** Allele frequencies of polymorphisms in FC27-like *msp2* of *P*. *falciparum* isolates from mild, severe and cerebral malaria patients in Thailand.

Region	Polymorphic position^a^		Mild	Severe ((S)(S)	Cerebral	Total	M vs S^d^	M vs C	S vs C
	Nucleotide	Codon (aa.)	(%)	(%)	(%)	(%)	*P*-value, OR	*P*-value,OR	*P*-value,OR
Block 2	23 A/C^b^	8 AAG (K)	19 (35.2)	7 (25.0)	13 (40.6)	39 (34.2)	0.347, 0.61	0.614, 1.26	0.200, 2.05
	**24 G/T**	**· · T (N)**	**13 (24.1)**	**13 (46.4)**	**7 (21.9)**	**33 (28.9)**	**0.039, 2.73**	0.816, 0.88	**0.044, 0.32**
		**·** CT (T)	22 (40.7)	8 (28.6)	12 (37.5)	42 (36.8)	0.278, 0.58	0.766, 0.87	0.464, 1.50
	27 T/G	9 AGT (S)	45 (83.3)	18 (64.3)	26 (81.3)	89 (78.1)	0.053, 2.78	0.806, 1.15	0.138, 0.42
		**· ·**G (R)	9 (16.7)	10 (35.7)	6 (18.8)	25 (21.9)			
	32 G/A	11 GGT (G)	51 (94.4)	25 (89.3)	31 (96.9)	107 (93.9)	NA.	NA.	NA.
		**·** A **·** (D)	3 (5.6)	3 (10.7)	1 (3.1)	7 (6.1)			
	37 A/G	13 AAT (N)	50 (92.6)	25 (89.3)	31 (96.9)	106 (93.0)	NA.	NA.	NA.
	39 T/A	**· ·** A (K)	3 (5.6)	3 (10.7)	1 (3.1)	7 (6.1)	NA.	NA.	NA.
		G**· ·** (D)	1 (1.9)	0 (0)	0 (0)	1 (0.9)	NA.	NA.	NA.
	48 A/T	16 AAA (K)	33 (61.1)	18 (64.3)	21 (65.6)	72 (63.2)	0.779, 0.87	0.676, 0.82	0.914, 0.94
		**· ·** T (N)	21 (38.9)	10 (35.7)	11 (34.4)	42 (36.8)			
	49_57indel	17_19 del	53 (98.1)	26 (92.9)	32 (100.0)	111 (97.4)	NA.	NA.	NA.
		17_19 ins GCT CCA AAA (APK)	0 (0)	2 (7.1)	0 (0)	2 (1.8)	NA.	NA.	NA.
		17_19 ins GCT CCA AAT (APN)	1 (1.9)	0 (0)	0 (0)	1 (0.9)	NA.	NA.	NA.
Block3^c^	**(R1)**_**n**_(R2)_n_	***(R1)***(R2)(R2)	1 (1.9)	2 (7.1)	0 (0)	3 (2.6)	NA.	NA.	NA.
		***(R1)***(R2)(R2)(R2)	39 (72.2)	22 (78.6)	21 (65.6)	82 (71.9)	0.532, 1.41	0.520, 0.73	0.267, 0.52
		***(R1)***(R2)(R2)(R2)(R2)	7 (13.0)	3 (10.7)	9 (28.1)	19 (16.7)	0.768, 0.81	0.081, 2.63	0.093, 3.26
		***(R1)***(R2)(R2)(R2)(R2)(R2)	5 (9.3)	1 (3.6)	1 (3.1)	7 (6.1)	NA.	NA.	NA.
		***(R1)(R1)(R1)***(R2)	2 (3.7)	0 (0)	1 (3.1)	3 (2.6)	NA.	NA.	NA.
	R1xR2	A 12	1 (1.9)	0 (0)	0 (0)	1 (0.9)	NA.	NA.	NA.
Block 3^c^		A 122	20 (37.0)	15 (53.6)	11 (34.4)	46 (40.3)	0.151, 1.96	0.804, 0.89	0.134, 0.45
		**A 1222**	**6 (11.1)**	**0 (0)**	**7 (21.9)**	**13 (11.4)**	0.066	0.178, 2.24	**0.009**
		A 132	2 (3.7)	1 (3.6)	0 (0)	3 (2.6)	NA.	NA.	NA.
		A 1333	0 (0)	2 (7.1)	0 (0)	2 (1.8)	NA.	NA.	NA.
		A 222	3 (5.6)	0 (0)	0 (0)	3 (2.6)	NA.	NA.	NA.
		A 333	14 (25.9)	6 (21.4)	10 (31.3)	30 (26.3)	0.653, 0.78	0.595, 1.30	0.391, 1.67
		A 3333	0 (0)	0 (0)	2 (6.3)	2 (1.8)	NA.	NA.	NA.
		A 33333	5 (9.3)	1 (3.6)	1 (3.1)	7 (6.1)	NA.	NA.	NA.
		B 22	0 (0)	2 (7.1)	0 (0)	2 (1.8)	NA.	NA.	NA.
		B 2222	0 (0)	1 (3.6)	0 (0)	1 (0.9)	NA.	NA.	NA.
		C 2111	1 (1.9)	0 (0)	0 (0)	1 (0.9)	NA.	NA.	NA.
		ADD 2	2 (3.7)	0 (0)	1 (3.1)	3 (2.6)	NA.	NA.	NA.
Block 4	58 G/A	20 GAA (E)	43 (79.6)	24 (85.7)	30 (93.8)	97 (85.1)	0.499, 0.65	0.077, 0.26	0.301, 0.4
		A **· ·** (K)	11 (20.4)	4 (14.3)	2 (6.3)	17 (14.9)			
	64 C/A	22 CAA (Q)	44 (81.5)	24 (85.7)	30 (93.8)	98 (86.0)	0.629, 0.73	0.113, 0.29	0.301, 0.4
		A **· ·** (K)	10 (18.5)	4 (14.3)	2 (6.3)	16 (14.0)			

^a^ Position relative to the first nucleotide / aa. of each block (Fig 2).

^b^ In case of SNPs, alleles found in the *msp2* sequence of K1 (FC27-liked) (M59766.1) / another found in our data set was shown and amino acid (aa.) changes were indicated.

^c^ Variation in number of repeat 1 and 2 [(R1)_n_(R2)_n_] generated 5 distinct alleles in block 3, while 13 alleles were detected when sequence variation in repeat units were considered [R1xR2].

^d^ Allele frequencies were compared between mild (M) and severe (S), mild and cerebral (C), as well as severe and cerebral. For bi-allelic polymorphisms, the odds ratio (OR) of a minor-frequency allele for risk to severe and cerebral malaria by comparing to a major allele was analyzed. For polymorphisms with more than 2 alleles, the presence or absence of individual alleles were compared. OR and *P*-values are shown, with significant values in bold. NA. (not applicable) indicates bi-allelic polymorphisms with minor allele frequency <10% and individual alleles having frequencies <10% or >90%, in which their associations with malaria severity were not analyzed. OR was undefined in cases of zero cell count.

**Table 4:** Haplotype frequencies of *P*. *falciparum* FC27-like *msp2* of from mild, severe and cerebral malaria patients in Thailand, comprising polymorphisms in block 2, 3 and 4.

FC27 haplotype	Amino acid changes^a^									Mild (%)	Severe (%)	Cerebral (%)	Total (%)	M vs S^c^ *P*-value, OR	M vs C *P*-value, OR	S vs C *P*-value, OR
BI.2 - BI.3 - BI.4 Haplotype 1^b^	8	-9	-11	-13	-16	-indel	-R1R2	-20	-22	14 (25.9)	6 (21.4)	10 (31.3)	30 (26.3)	0.653, 0.78	0.595, 1.30	0.39, 1.67
	K	S	G	N	K	del	A333	E	Q							
2	**·**	**·**	**·**	**·**	**·**	del	A33333	**·**	**·**	0 (0)	0 (0)	2 (6.3)	2 (1.8)	NA.	NA.	NA.
3	**·**	**·**	**·**	**·**	**·**	del	A33333	**·**	**·**	5 (9.3)	1 (3.6)	1 (3.1)	7 (6.1)	NA.	NA.	NA.
4	**T**	**·**	**·**	**·**	**·**	del	A122	**·**	**·**	3 (5.6)	0 (0)	2 (6.3)	5 (4.4)	NA.	NA.	NA.
5	T	**·**	**·**	**·**	**N**	del	A122	**·**	**·**	1 (1.9)	3 (10.7)	1 (3.1)	5 (4.4)	NA.	NA.	NA.
**6**^b^	**T**	**·**	**·**	**·**	**N**	del	A1222	**·**	**·**	**5 (9.3)**	**0 (0)**	**7 (21.9)**	**12 (10.5)**	0.097	0.103, 2.75	**0.009**
7	T	**·**	**·**	**·**	N	del	A132	**·**	**·**	2 (3.7)	1 (3.6)	0 (0)	3 (2.6)	NA.	NA.	NA.
8	T	**·**	**·**	**·**	N	del	A1222	K	**·**	1 (1.9)	0 (0)	0 (0)	1 (0.9)	NA.	NA.	NA.
9	T	**·**	**·**	**·**	N	del	A12	K	K	1 (1.9)	0 (0)	0 (0)	1 (0.9)	NA.	NA.	NA.
10^b^	T	**·**	**·**	**·**	N	del	A122	K	K	8 (14.8)	4 (14.3)	2 (6.3)	14 (12.3)	0.949, 0.96	0.231, 0.38	0.301, 0.4
11	T	R	**·**	**·**	N	del	A122	**·**	**·**	1 (1.9)	0 (0)	0 (0)	1 (0.9)	NA.	NA.	NA.
12	N	·	**·**	**·**	N	APN	A222	K	K	1 (1.9)	0 (0)	0 (0)	1 (0.9)	NA.	NA.	NA.
13	N	·	**·**	D	**·**	del	C2111	·	·	1 (1.9)	0 (0)	0 (0)	1 (0.9)	NA.	NA.	NA.
14	N	·	D	K	**·**	del	ADD2	·	·	1 (1.9)	0 (0)	0 (0)	1 (0.9)	NA.	NA.	NA.
15	N	·	D	K	**·**	del	B22	·	·	0 (0)	2 (7.1)	0 (0)	2 (1.8)	NA.	NA.	NA.
16	N	·	D	K	**·**	del	A222	·	·	1 (1.9)	0 (0)	0 (0)	1 (0.9)	NA.	NA.	NA.
17	N	·	D	K	**·**	del	B2222	·	·	0 (0)	1 (3.6)	0 (0)	1 (0.9)	NA.	NA.	NA.
18	N	·	D	K	N	del	ADD2	·	·	1 (1.9)	0 (0)	1 (3.1)	2 (1.8)	NA.	NA.	NA.
19^b^	N	R	·	·	·	del	A122	·	·	7 (13.0)	8 (28.6)	6 (18.8)	21 (18.4)	0.083, 2.69	0.469, 1.55	0.370, 0.58
20	N	R	·	·	·	del	A222	·	·	1 (1.9)	0 (0)	0 (0)	1 (0.9)	NA.	NA.	NA.
21	N	R	·	·	N	APK	A1333	·	·	0 (0)	2 (7.1)	0 (0)	2 (1.8)	NA	NA.	NA.

^a^ Position relative to the first aa. of each block (Fig 2).

^b^ Major haplotypes (frequency ≥ 10%) observed in the parasite population that were analyzed for association with malaria severity.

^c^ Haplotype frequencies were compared between mild (M) and severe (S), mild and cerebral (C), and severe and cerebral.

*P*-value and Odds ratios (OR) are shown, with statistically significant differences in bold. NA. (not applicable) indicates haplotypes with frequencies >10% whose association with malaria severity was not analyzed. OR was undefined in cases of zero cell count.

**Fig 3:**
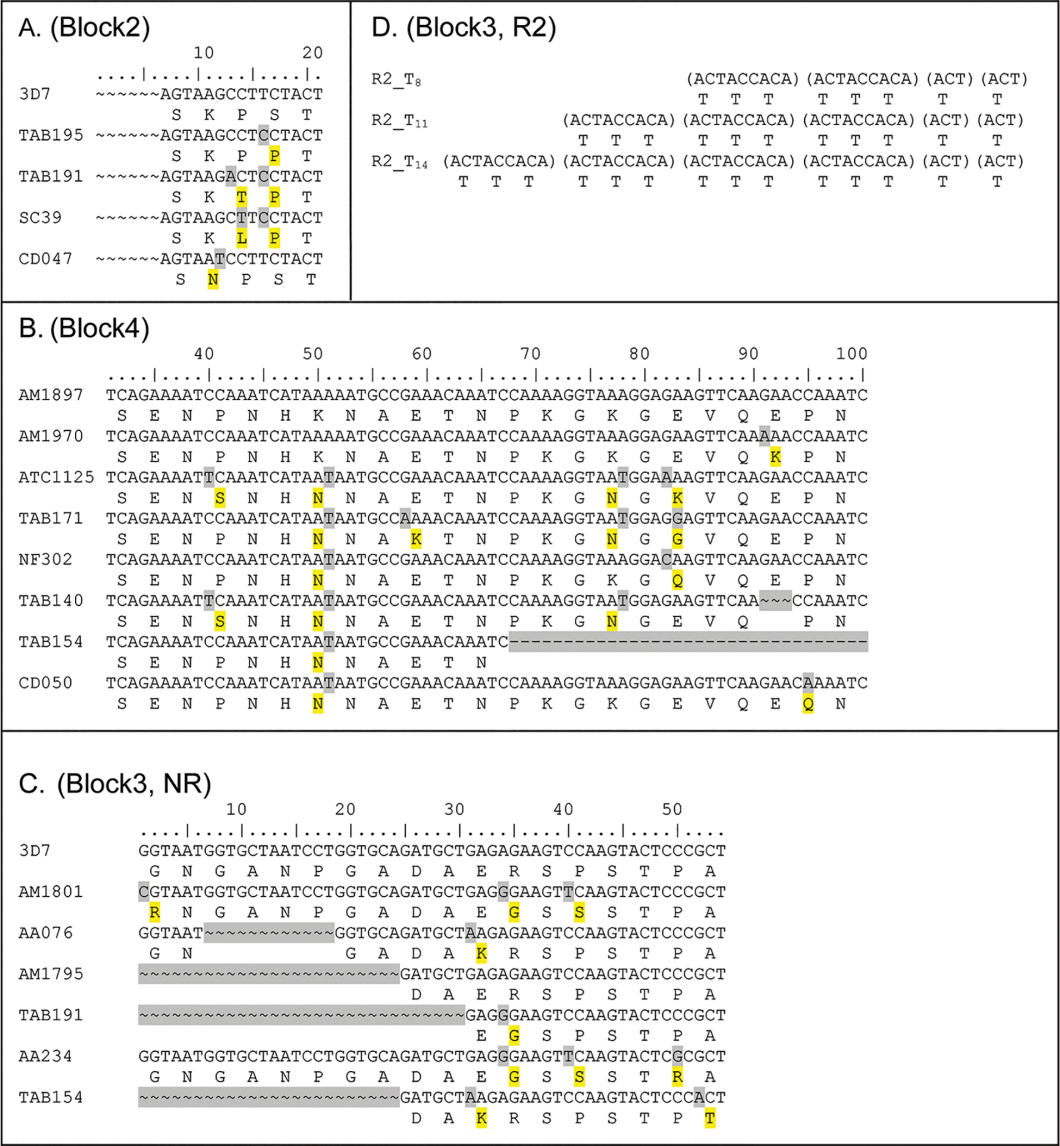
Sequence alignment of *Msp2* 3D7 family of *Plasmodium falciparum* showing polymorphisms in each block. Changes in nt. / aa. are shaded gray / yellow. The *Msp2* sequence for 3D7 (GenBank accession number: PFB0300c) was used as a reference for alignment of Block 2, 4, and the non-repetitive region (NR) of block 3. (A) Block 2: first 6 nt. residues of 21 (7 aa.) are not analyzed. Four non-synonymous SNPs are shown. (B) Block 4: only residues from nt. 31 to 100 of 270 (90 aa.) are presented. Eight non-synonymous SNPs and 2 indel are shown. (C) Block 3, non-repeat region (NR): all 54 nt. (18 aa.) are presented, showing 6 non-synonymous SNPs and 3 indel. (D) Block 3, repeat region 2 (R2): 2, 3, and 4 copies of nanomer (ACT ACC ACA) followed by ACT ACT producing Threonine 8, 11, and 14 residues, respectively.

For the 3D7 family, nucleotide alignment of 163 3D7-liked *msp2* sequences demonstrated more diverse and complex sequences than those for FC27. Several non-synonymous SNPs and indel were found in block 2, 4 and the NR region of block 3 (Fig 3A, 3B and 3C and Table 5). In block 2, four non-synonymous SNPs were found while in block 4, eight non-synonymous SNPs and 2 indel were found. In the NR of block 3, six non-synonymous SNPs and 3 indel were found. R1 of block 3 contained extremely diverse sequences—mostly Glycine (G), Serine (S), and Alanine (A). Because of significant diversity, alignment of repeat sequences in R1 was difficult. According to the previous study that considered the amino acids GA dipeptide (encoded by GGT GCT) as the ancestral repeat [32, 33], we aligned the R1 region by amino acid. Ten different dipeptide motifs (GA, GS, GG etc.) were observed (coded 0–9 in S1 Table). Based on the motif arrangement, 54 distinct alleles were found in this region that can be grouped into nine types (162, 185, 18585, etc.) according to the presence of different types of motif sequences (S1 Table). In R2, we found 8 (T_8_), 11 (T_11_), and 14 (T_14_) Threonine repeats that were encoded by 2–4 copies of nanomer (ACT ACC ACA) followed by ACT ACT (Fig 3D, Table 5). Overall, combining polymorphisms in block 2, 3, and 4 generated 62 distinct haplotypes among 163 3D7-like *msp2* strains (GenBank accession numbers JX8858919-JX885980). Haplotype analysis of polymorphisms in each block, except for the R1 region are shown in Table 6.

**Table 5:** Allele frequencies of polymorphisms in the 3D7-like *msp2* sequences of *P*. *falciparum* isolates from mild, severe and cerebral malaria patients in Thailand.

Region	Polymorphic position^a^		Mild	Severe	Cerebral	Total	M vs S^e^	M vs C	S vs C
	Nucleotide	Codon (aa.)	(%)	(%)	(%)	(%)	*P*-value, OR	*P*-value, OR	*P*-value, OR
Block 2	12 G/T^b^	4 AAG (K)	26 (43.3)	24 (45.3)	16 (37.2)	66 (42.3)	0.835, 1.08	0.533, 0.77	0.425, 0.72
		**· ·** T (N)	34 (56.7)	29 (54.7)	27 (62.8)	90 (57.7)			
	13 C/A	5 CCT (P)	48 (78.7)	47 (83.9)	36 (83.7)	131 (81.9)	0.469, 1.41	0.521, 1.39	0.978, 0.98
	14 C/T	A **· ·** (T)	11 (18.0)	8 (14.3)	7 (16.3)	26 (16.3)	0.583, 0.76	0.816, 0.88	0.784, 1.17
		**·** T **·** (L)	2 (3.3)	1 (1.8)	0 (0)	3 (1.9)	NA.	NA.	NA.
	16 T/C	6 TCT (S)	7 (11.5)	7 (12.5)	7 (16.3)	21 (13.1)	0.865, 1.10	0.480, 1.50	0.593, 1.36
		C **· ·** (P)	54 (88.5)	49 (87.5)	36 (83.7)	139 (86.9)			
Block 3	**R1 region** ^**c**^	162: GAVAGS	14 (23.0)	11 (19.6)	11 (23.9)	36 (22.1)	0.663, 0.82	0.907, 1.06	0.602, 1.29
		185: GASGSA	10 (16.4)	8 (14.3)	8 (14.3)	26 (16.0)	0.752, 0.85	0.891, 1.07	0.668, 1.26
		1852: GASGSAGS	7 (11.5)	13 (23.2)	5 (10.9)	25 (15.3)	0.092, 2.33	0.922, 0.94	0.104, 0.40
		18585: GASGSASGSA	6 (9.8)	6 (10.7)	6 (13.0)	18 (11.0)	0.876, 1.11	0.603, 1.38	0.716, 1.25
		2165: GSGAVASA	6 (9.8)	3 (5.4)	2 (4.3)	11 (6.7)	NA.	NA.	NA.
		27165: GSRDGAVASA	6 (9.8)	6 (10.7)	2 (4.3)	14 (8.6)	NA.	NA.	NA.
		35: GGSA	5 (8.2)	2 (3.6)	4 (8.7)	11 (6.7)	NA.	NA.	NA.
		385: GGSGSA	6 (9.8)	7 (12.5)	8 (17.4)	21 (12.9)	0.647, 1.31	0.251, 1.93	0.488, 1.47
		385_35: GGSGSA GGSA	1 (1.6)	0 (0)	0 (0)	1 (0.6)	NA.	NA.	NA.
	**NR region**								
	1_30 indel	1_10 ins GNGANPGADA	18 (29.5)	12 (21.4)	13 (28.3)	43 (26.4)	0.317, 0.65	0.888, 0.94	0.425, 1.44
		**1_10 ins** **R****NGANPGADA**	**13 (21.3)**	**19 (33.9)**	**7 (15.2)**	**39 (23.9)**	0.126, 1.90	0.423, 0.66	**0.031, 0.35**
		3_6 del GN---- GADA	14 (23.0)	11 (19.6)	11 (23.9)	36 (22.1)	0.663, 0.82	0.907, 1.06	0.602, 1.29
		1_8 del- - - - - - - - DA	9 (14.8)	6 (10.7)	9 (19.6)	24 (14.7)	0.514, 0.69	0.510, 1.41	0.209, 2.03
		1_10 del- - - - - - - - - -	7 (11.5)	8 (14.3)	6 (13.0)	21 (12.9)	0.650, 1.29	0.806, 1.16	0.856, 0.90
	31 G/A	11 GAG (E)	46 (75.4)	44 (78.6)	33 (71.7)	23 (75.5)	0.685, 0.84	0.669, 1.21	0.425, 1.44
		A **· ·** (K)	15 (24.6)	12 (21.4)	13 (28.3)	40 (24.5)			
	34 A/G	12 AGA (R)	35 (57.4)	30 (53.6)	31 (67.4)	96 (58.9)	0.679, 1.17	0.292, 0.65	0.157, 0.56
		G **· ·** (G)	26 (42.6)	26 (46.4)	15 (32.6)	67 (41.1)			
	40 C/T	14 CCA (P)	41 (67.2)	35 (62.5)	37 (80.4)	113 (69.3)	0.594, 1.23	0.128, 0.50	**0.048, 0.41**
		**T · · (S)**	**20 (32.8)**	**21 (37.5)**	**9 (19.6)**	**50 (30.7)**			
	50 C/G	17 CCC (P)	60 (98.4)	56 (100.0)	44 (95.7)	160 (98.2)	NA.	NA.	NA.
		**·** G **·** (R)	1 (1.6)	0 (0)	2 (4.3)	3 (1.8)			
	52 G/A	18 GCT (A)	61 (100)	56 (100)	45 (97.8)	162 (99.4)	NA.	NA.	NA.
		A **· ·** (T)	0 (0)	0 (0)	1 (2.2)	1 (0.6)			
	**R2 region**^**d**^								
	(ACT ACC ACA)_2_ ACT_2_ ACT	(T)_8_	42 (68.9)	37 (66.1)	35 (76.1)	114 (69.9)	0.748, 0.88	0.410, 1.44	0.269, 1.63
	(ACT ACC ACA)_3_ ACT_2_ ACT	(T)_11_	8 (13.1)	4 (7.1)	7 (15.2)	19 (11.7)	0.288, 0.51	0.757, 1.19	0.191, 1.33
	**(ACT ACC ACA)**_**4**_ ACT_2_ ACT	**(T)**_**14**_	**11 (18.0)**	**15 (26.8)**	**4 (8.7)**	**30 (18.7)**	0.255, 1.66	0.168, 0.43	**0.020, 0.26**
Block 4	40 C/T	14 CCA (P)	55 (90.2)	50 (89.3)	44 (95.7)	149 (91.4)	NA.	NA.	NA.
		T **· ·** (S)	6 (9.8)	6 (10.7)	2 (4.3)	14 (8.6)			
	**51 A/T**	**17 AAA (K)**	**5 (8.2)**	**7 (12.5)**	**11 (23.9)**	**23 (14.1)**	0.443, 1.60	**0.024, 3.52**	0.132, 2.20
		**· ·** T (N)	56 (91.8)	49 (87.5)	35 (76.1)	140 (85.9)			
	58 G/A	20 GAA (E)	38 (62.3)	39 (69.6)	31 (67.4)	108 (66.3)	0.403, 0.72	0.586, 0.80	0.807, 1.11
		A **· ·** (K)	23 (37.7)	17 (30.4)	15 (32.6)	55 (33.7)			
	78 A/T	26 AAA (K)	30 (53.6)	35 (64.8)	28 (68.3)	93 (61.6)	0.231, 0.62	0.144, 0.54	0.722, 0.86
		**· ·** T (N)	26 (46.4)	19 (35.2)	13 (31.7)	58 (38.4)			
	82 G/C/A	28 GAA (E)	29 (51.8)	34 (63.0)	27 (65.9)	90 (59.6)	0.236, 1.58	0.166, 1.80	0.771, 1.13
	83 A/G	**·** G **·** (G)	17 (30.4)	12 (22.2)	10 (24.4)	39 (25.8)	0.333, 0.66	0.517, 0.74	0.804, 1.13
		C **· ·** (Q)	7 (12.5)	4 (7.4)	2 (4.9)	13 (8.6)	NA.	NA.	NA.
		A **· ·** (K)	3 (5.4)	4 (7.4)	2 (4.9)	9 (6.0)	NA.	NA.	NA.
	91_93 indel	31 ins GAA (E)	26 (46.4)	27 (50.0)	15 (36.6)	68 (45.0)	0.867, 1.07	0.078, 2.08	0.110, 1.95
		ins AAA (K)	24 (42.9)	24 (44.4)	25 (61.0)	73 (48.3)	0.837, 1.08	0.332, 0.67	0.242, 0.62
		del	6 (10.7)	3 (5.6)	1 (2.4)	10 (6.6)	NA.	NA.	NA.
	95 C/A	32 CCA (P)	51 (91.1)	43 (79.6)	37 (90.2)	131 (86.8)	0.089, 2.61	0.890, 1.10	0.160, 0.42
		**·** A **·** (Q)	5 (8.9)	11 (20.4)	4 (9.8)	20 (13.2)			
	67_99 indel	23_33 insert	56 (91.8)	54 (96.4)	41 (89.1)	151 (92.6)	NA.	NA.	NA.
		23_33 deletion	5 (8.2)	2 (3.6)	5 (10.9)	12 (7.4)			

^a^ Position relative to the first nucleotide / aa. of each block (Fig 3)

^b^ In case of SNPs, alleles found in the *msp2* sequence of 3D7 (PFB0300c) / another allele found in our data set was shown, and amino acid (aa.) changes are indicated.

^c^ For The R1 region in block 3, sequences can be grouped into nine types according to the presence of different types of numerically coded dipeptide motifs (S1 Table).

^d^ For the R2 region, there were 8, 11, and 14 Threonine repeats encoded by 2–4 copies of nanomer (ACT ACC ACA) followed by ACT ACT.

^e^ Allele frequencies were compared between mild (M) and severe (S), mild and cerebral (C), and severe and cerebral. For bi-allelic polymorphisms, the odds ratios (OR) of minor-frequency alleles compared to major alleles associated with severe and cerebral malaria were analyzed. For polymorphisms with more than 2 alleles, the presence/absence of individual alleles were compared. OR and *P*-values are shown, with significant differences in bold. NA. (not applicable) indicates bi-allelic polymorphisms with a minor allele frequency <10% and individual alleles with frequencies <10% or >90%, in which their association with malaria severity were not analyzed.

**Table 6:** Haplotype frequencies of 3D7 like *msp2* of *P*. *falciparum* from mild, severe and cerebral malaria patients in Thailand, with each block analyzed separately.

3D7 haplotype	Amino acid changes^a^	Mild (%)	Severe (%)	Cerebral (%)	Total (%)	M vs S^c^ *P*-value, OR	M vs C^c^ *P*-value, OR	S vs C^c^ *P*-value, OR
**Block 2**	4–5–6							
Haplotype 1^b^	N P P	27 (45.0)	22 (41.5)	20 (46.5)	69 (44.2)	0.709, 0.87	0.879, 1.06	0.623, 1.23
2^b^	**· ·** S	7 (11.7)	7 (13.2)	7 (16.3)	21 (13.5)	0.804, 1.15	0.501, 1.47	0.672, 1.28
3^b^	K **· ·**	13 (21.7)	17 (32.1)	9 (20.9)	39 (25.0)	0.211, 1.71	0.928, 0.96	0.222, 0.56
4^b^	K T **·**	11 (18.3)	6 (11.3)	7 (16.3)	24 (15.4)	0.298, 0.57	0.787, 0.87	0.480, 1.52
5	K L **·**	2 (3.3)	1 (1.9)	0 (0)	3 (1.9)	NA.	NA.	NA.
**Block 3** NR-R2	1_10indel -11–12–14–17–18 - [T]							
Haplotype 1^b^	Ins G E R P P A 8	11 (18.0)	8 (14.3)	10 (21.7)	29 (17.8)	0.583, 0.76	0.633, 1.26	0.326, 1.67
2	Ins G **·** G S **· ·** 14	4 (6.6)	3 (5.4)	0 (0)	7 (4.3)	NA.	NA.	NA.
3	Ins G **·** G S **· ·** 11	1 (1.6)	0 (0)	0 (0)	1 (0.6)	NA.	NA.	NA.
4	Ins G **·** G S R **·** 11	1 (1.6)	0 (0)	2 (4.3)	3 (1.8)	NA.	NA.	NA.
5	Ins G K **· · · ·** 8	1 (1.6)	1 (1.8)	1 (2.2)	3 (1.8)	NA.	NA.	NA.
6	Ins R **· · · · ·** 11	0 (0)	1 (1.8)	0 (0)	1 (0.6)	NA.	NA.	NA.
7	Ins R **·** G S **· ·** 8	6 (9.8)	6 (10.7)	2 (4.3)	14 (8.6)	NA.	NA.	NA.
8^b^	Ins R **·** G S **· ·** 14	6 (9.8)	12 (21.4)	4 (8.7)	22 (13.5)	0.083, 2.50	0.841, 0 .87	0.079, 0.35
9	Ins R **·** G S **· ·** 11	1 (1.6)	0 (0)	1 (2.2)	2 (1.2)	NA.	NA.	NA.
10^b^	Del3_6 K **· · · ·** 8	14 (23.0)	11 (19.6)	11 (23.9)	36 (22.1)	0.663, 0.82	0.907, 1.06	0.602, 1.29
11	Del1_8 **· · · · ·** 8	4 (6.6)	6 (10.7)	4 (8.7)	14 (8.6)	NA.	NA.	NA.
12	Del1_8 **· · · · ·** 11	5 (8.2)	3 (5.4)	4 (8.7)	12 (7.4)	NA.	NA.	NA.
13	Del1_8 **·** G S **· ·** 14	1 (1.6)	0 (0)	0 (0)	1 (0.6)	NA.	NA.	NA.
14	Del1_8 K **· · ·** T 8	0 (0)	0 (0)	1 (2.2)	1 (0.6)	NA.	NA.	NA.
15^b^	Del1_10 **·** G **· · ·** 8	6 (9.8)	5 (8.9)	6 (13.0)	17 (10.4)	0.867, 0.90	0.603,1.38	0.505, 1.53
**Block 4**	14–17–20–26–28–31–32							
Haplotype 1^b^	P N E K E K P	7 (11.5)	8 (14.3)	6 (13.0)	21 (12.9)	0.650, 1.29	0.806, 1.16	0.856, 0.90
2^b^	**· · · · ·** E Q	5 (8.2)	11 (19.6)	4 (8.7)	20 (12.3)	0.072, 2.74	0.927, 1.07	0.120, 0.39
3	**· · · ·** Q E **·**	7 (11.5)	4 (7.1)	2 (4.3)	13 (8.0)	NA.	NA.	NA.
4	**· · ·** N K **· ·**	1 (1.6)	0 (0)	0 (0)	1 (0.6)	NA.	NA.	NA.
5	**· · ·** N K E **·**	2 (3.3)	1 (1.8)	1 (2.2)	4 (2.5)	NA.	NA.	NA.
6	**· ·** K **· · · ·**	6 (9.8)	5 (8.9)	5 (10.9)	16 (9.8)	NA.	NA.	NA.
7	**· ·** K N G **· ·**	5 (8.2)	4 (7.1)	3 (6.5)	12 (7.4)	NA.	NA.	NA.
8^b^	**· ·** K N G E **·**	12 (19.7)	8 (14.3)	7 (15.2)	27 (16.6)	0.439, 0.68	0.551, 0.73	0.895, 1.08
**9**^b^	**· K · · · · ·**	**5 (8.2)**	**7 (12.5)**	**11 (23.9)**	**23 (14.1)**	0.443, 1.60	**0.024, 3.52**	0.132, 2.20
10	S **· ·** N **·** del **·**	6 (9.8)	3 (5.4)	1 (2.2)	10 (6.1)	NA.	NA.	NA.
11	S **· ·** N K **· ·**	0 (0)	3 (5.4)	1 (2.2)	4 (2.5)	NA.	NA.	NA.
12	**· · ·** ---- 23_33 del----	5 (8.2)	2 (3.6)	5 (10.9)	12 (7.4)	NA.	NA.	NA.

^a^ Position relative to the first aa. of each block (Fig 3).

^b^ Major haplotypes (frequency ≥ 10%) observed in the parasite population that were analyzed for association with malaria severity.

^c^ Haplotype frequencies were compared between mild (M) and severe (S), mild and cerebral (C), as well as severe and cerebral. *P*-values and odds ratios (OR) are shown, with significant differences in bold. NA. (not applicable) indicates haplotypes with frequencies >10% whose associations with malaria severity were not analyzed.

### Association of *msp2* with malaria severity

Frequency distributions of FC27 and 3D7-like *msp2* based on malaria severity are shown in Table 2. No significant differences were observed. Table 3 and Table 5 show allele frequencies of polymorphisms in the FC27 and 3D7-like *msp2* sequences of *P*. *falciparum* isolates from mild, severe and cerebral malaria patients. When allele frequenices in the severe and mild malaria groups were compared, a significantly higher frequency of the block 2-8N alelle of FC27-like *msp2* was found in patients with severe malaria (46.4%) compared to mild malaria (24.1%) with an odds ratio of 2.73 (*P* = 0.039). No significant difference was detected for alleles of 3D7-like *msp2*. However, when comparing the cerebral and mild malaria groups, a significant 2-fold higher frequency of the K allele was found for block 4-17K/N of 3D7-like *msp2* in patients with cerebral malaria (23.9%) compared to mild malaria (12.5%). The odds ratio for the K allele in patients with cerebral malaria was 3.52 (*P* = 0.024). These findings suggest that block 2-8N of FC27 family and block4-17K of the 3D7 family may represent virulent genotypes for severe, and cerebral malaria, respectively. Moreover, bias in allele frequency distribution between severe and cerebral malaria was also observed for block 2-8N allele of FC27 family, as well as other loci in FC27 and 3D7–like *msp2* (Tables 3 and 5).

Given several polymorphisms detected in *msp2*, relevant *msp2* haplotypes were also analyzed for their association with malaria severity. For FC27-like *msp2*, twenty-one distinct haplotypes containing all polymorphisms of block 2, 3, and 4 were observed among 114 samples with FC27-like *msp2* (Table 4). Of these, four major haplotypes with frequencies ≥ 10% (haplotypes 1, 6, 10, and 19) were found and analyzed. No differences in frequency were detected between mild vs. severe malaria or mild vs. cerebral malaria. However, a significantly increased frequency of haplotype 6 was found in patients with cerebral malaria (21.9%), compared to absence of this haplotype in severe malaria (*P* = 0.09). For 3D7-like *msp2* which exhibited much greater diversity, haplotypes of each block were analyzed separately. For block 3, haplotypes contained only the NR and R2 region. R1 was not included given large variation (54 types) which obviated meaningful comparisons. Distinct haplotypes of 5, 15 and 12 were identified for block 2, 3, and 4, respectively (Table 6). We found a significant difference in the frequency of block 4 haplotype 9 (PKEKEKP) between mild and cerebral malaria. This haplotype was associated with cerebral malaria (OR = 3.52, *P* = 0.02). The apparent association seems to come from the presence of the block 4-17K allele in this haplotype since the *P* value and OR were equal to those obtained for the block 4-17K allele alone.

### LD structure of *msp2*

**Fig 4:**
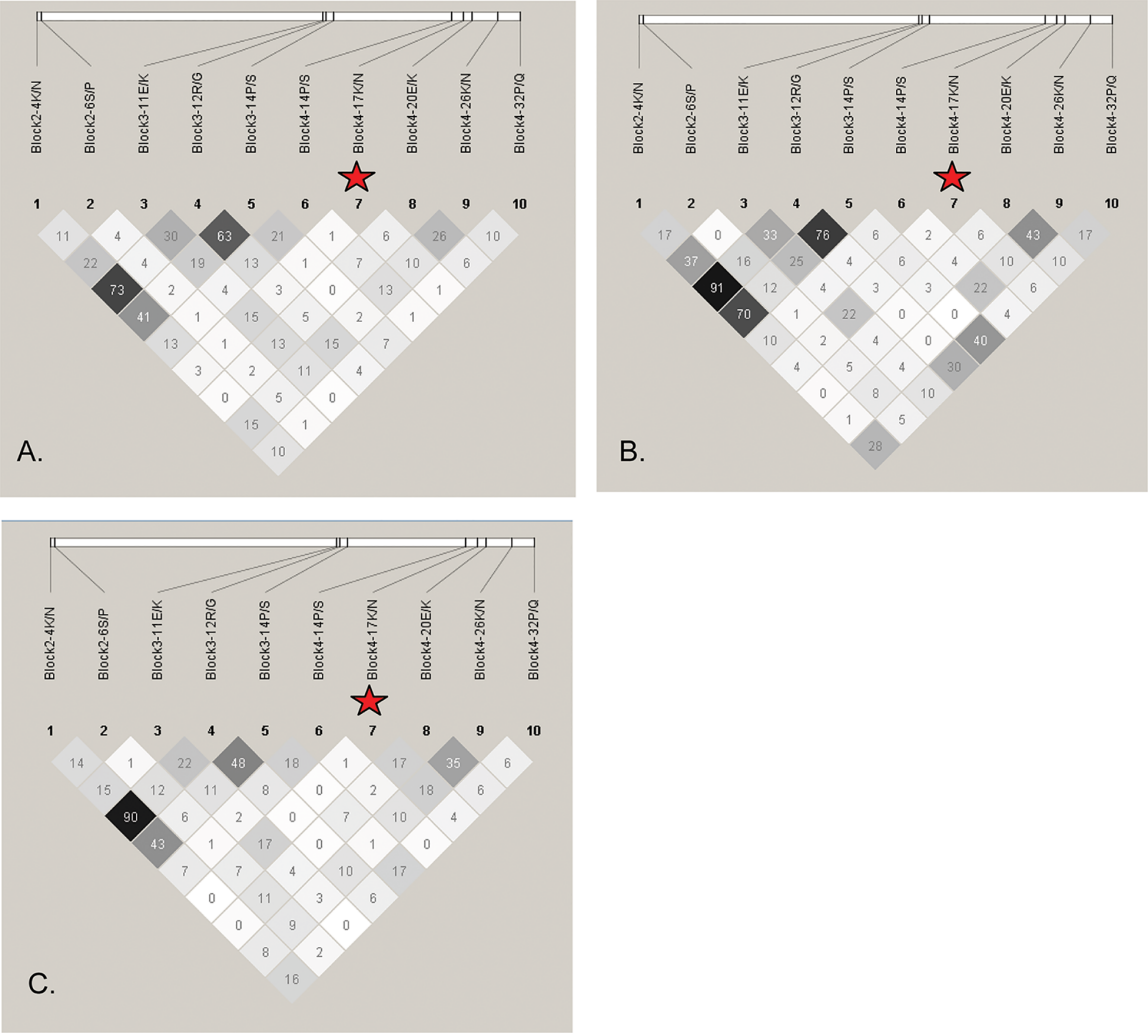
Linkage disequilibrium (LD) structures of 3D7-liked *msp2* of *P*. *falciparum* in Thailand. Pairwise LD plots based on r^2^ between the 10 bi-allelic polymorphisms with minor allele frequency ≥ 10% were calculated for *P*. *falciparum* from mild (A), severe (B), and cerebral malaria patients (C). White, shades of grey, and black squares indicate no LD (r^2^ = 0), intermediate LD (0 < r^2^ < 1), and strong LD (r^2^ = 1), respectively. LD structures were plotted using haploview software and amino acid changes are shown. The polymorphisms associated with cerebral malaria are labeled with a red star.

Pairwise LD structure between the 10 bi-allelic polymorphisms with minor allele frequency ≥ 10% in 3D7-like *msp2* sequences was analyzed based on r^2^ values (Fig 4). There was not a noticeable difference in LD structure between parasites from the mild, cerebral and severe malaria groups. This suggests that differences in allele frequencies did not affect the LD profile of malaria parasites. Since no LD was found between 3D7 block 4-17N/K and other polymorphisms, the association of the block 4-17K allele with cerebral malaria is unlikely to be caused by LD from other polymorphisms. LD between FC27 block 2-8K/N/T which associated with severe malaria and other loci could not be analyzed, since this associated locus is a multi-allelic polymorphism. Therefore, analysis of LD structure for FC27-like *msp2* sequences was omitted.

### Association of *msp2* with parasitemia

To clarify the contribution of virulence-associated alleles to disease progression of severe and cerebral malaria, their associations with parasitemia were analyzed. No association was detected for block 2-8K/N/T of FC27-like *msp2* and block 4-17N/K of 3D7-like *msp2*. There were not significant differences in parasitemia between patients infected with different parasites genotypes (median parasitemia = 24,150, 26,250, and 25,380/μL for FC27 block 2-8K, N, T allele, respectively, *P*-value = 0.462; median parasitemia = 31,540 and 50,900 /μL for 3D7 block 4-17N and K, respectively, *P*-value = 0.563).

## Discussion

Parasite virulence is thought to be responsible for severity of *P*. *falciparum* infection. Although several studies have tried to characterize virulent strains using polymorphic genes as markers, they analyzed only size variation and bi-allelic families of genes which is insufficient to identify suitable virulence markers [2, 3, 5, 6, 24–27]. Therefore, conclusive virulence markers have not been described to date. In this study, we analyzed sequence variations of the most polymorphic merozoite surface protein, MSP2, in detail, and found the *msp2*_FC27_block 2-8N allele and 3D7_block 4-17K allele associated with severe and cerebral malaria, respectively, in Thailand. Lack of linkage disequilibrium between the associated alleles and other polymorphisms in *msp2* indicated that the association is independent of other polymorphisms. This is the first study to reveal allelic sequences that could be potential markers for severe and cerebral malaria. In addition to virulence-associated alleles, interestingly, a number of polymorphic loci including severe malaria associated locus FC27_block 2-8N showed differences in allele frequencies between severe and cerebral malaria. This implies that parasite genetic factors responsible for cerebral malaria are distinct from those that cause severe symptoms involving other organs. Moreover, we found a clinical association of multiple infections with increased risk for non-cerebral severe malaria, but a much lower frequency of multiple infections observed in cerebral malaria patients. These findings correspond with previous studies where the epidemiological observations and parasite genetic characterization demonstrated the association of cerebral malaria with emergence of a few distinct virulent strains [1, 3, 8, 34].

While it remains unclear whether these associated alleles were the primary cause of virulence or just markers based on LD to a causal variant in another locus, their contribution to severe malaria pathogenesis is hypothesized. Amino acid changes from positively charged lysine (K) to an uncharged asparagine (N) or vice versa may have effects on protein function. Given *msp2*’s well characterized role in RBC invasion, the virulent allele is located in a specific RBC binding region [13] and may have an effect on invasion efficiency. Nonetheless, association with parasitemia was not detected. In addition to the role in parasite invasion, MSP2 is a target of naturally acquired clinical immunity to malaria [15, 16]. Antibodies induced by this antigen predominantly recognize its variable regions, and display strain-specific immunity [35]. A prior study showed that antibody responses to epitopes within the 3D7 dimorphic region have a protective role for malaria infection [36]. Virulence of associated alleles could be explained by the particular folding of the MSP2 protein that might contribute to the immune response. Immune-mediated pathogenicity of severe outcomes such as excessive production of certain cytokines has been raised [37, 38]. Thus, improper responses acquired by virulence-associated alleles may eventually cause severe or cerebral malaria.

Although the present study demonstrates the use of candidate gene association analysis to identify parasite genetic determinants of malaria severity, factors complicating study interpretation should be considered. Theoretically, when several polymorphic markers within a given candidate region are examined for association with a disease of interest, correction for multiple testing should be taken to account for spurious associations (type I error). However, multiple correction remains a problematic issue, especially for markers having several polymorphic loci or multiple alleles, as *msp2* [39]. By reducing the chance of type I error, the chance of type II error is increased, reducing the power to detect the true effect size of causal variants. Although this issue might be solved by increasing sample size, this would be difficult or impossible in practice due to the relatively rare occurrence of severe and cerebral malaria in Thailand and Southeast Asia. In addition, there is prior evidence for associations between *msp2* and virulence, and this served as the basis for our analysis to this gene. Therefore, type I error (false positive result) was less of a concern than type II error (false negative). Given these caveats, correction for multiple comparisons was omitted here, but replicating the study may be required to confirm our results [40, 41]. In addition to concerns with multiple comparisons, a spurious association may also be caused by parasite population stratification between groups with differing malaria outcomes. Genotyping at unlinked markers such as microsatellites in mitochondrial genes would help to better understand population stratification of samples. Absence of association with the unlinked markers would help to verify the significant associations of candidate markers seen here [41–43].

Here we discerned the limitations of employing *msp2* family and size variation to characterize parasite genetics associated with malaria severity. The study provided limited information on the dimorphic family and overall variant size of the gene. Sequence variations that might be related to disease severity were not revealed. We observed limited capacity of gel electrophoresis to differentiate distinct variants with similar length, as described previously [10]. Our analysis revealed sequence variations among same-sized variants. These suggests that genetic diversity based on *msp2* family and size variation is likely to be underestimated, and may explain inconclusive results from previous studies attempting to determine allelic variants based on family/size variation. Recently, massively parallel pyrosequencing tools have been used to characterize parasite diversity in individual infections [44]. This tool can identify uncommon variants, increasing resolution for studying parasite diversity. Next generation sequencing and genome-wide association analysis has become an important approach to uncover the genetic basis of malaria biology. Genome-wide patterns demonstrate evidence of drug or immune selection, helping to identify markers for antimalarial resistance and candidate genes for vaccine development [45–47]. Although genome-wide analysis is a high resolution tool to characterize the genetic complexity of *P*. *falciparum* within clinical infection [48, 49], the association with parasite virulence hasn’t been studied. It is likely that not only *msp2*, but other unidentified markers contribute to malaria pathogenesis. Genome-wide association analysis would be a powerful tool to discover other markers associated to malaria severity, and identify parasite factors which contribute to severe and cerebral malaria.

## Conclusion

The present study characterized sequence variants of *msp*2 in *P*. *falciparum* isolates from patients with different clinical presentations, suggesting that the K allele at codon17 of block 4 in 3D7 family and the N allele at codon8 of block 2 in FC27 family may be associated with increased risk for cerebral malaria and other severe complications, respectively. Differences in allele frequencies of several polymorphic loci between cerebral malaria and non-cerebral severe malaria implied parasite genetic factors responsible for cerebral malaria may be distinct from those that cause severe symptoms involving other organs. Their interactions with potential host factors for severe disease should also be explored. Functional study of this polymorphism may help to better understand parasite virulence leading to severe complications. This is the first study to our knowledge that identifies *msp*2 sequence polymorphisms as candidate virulence markers.

## Supporting information

S1 Fig Frequencies of different sized *msp2* variants determined by DNA sequencing (upper) and conventional gel electrophoresis (lower). Sequence analysis differentiated *msp2* variants into FC27 (red bars) or 3D7 (blue bars) families, respectively. The frequencies of variants sized by gel electrophoresis is shown by green bars in the lower panel.
(PDF)

S1 Table Genetic diversity of the R1 region in Block 3 of 163 3D7-liked *msp2* sequences. (PDF)
