# Correction: Sequence variation in *Plasmodium falciparum* merozoite surface protein-2 is associated with virulence causing severe and cerebral malaria

**DOI:** 10.1371/journal.pone.0196694

**Published:** 2018-04-25

**Authors:** Suwanna Chaorattanakawee, Pornlada Nuchnoi, Hathairad Hananantachai, Uranan Tumkosit, David Saunders, Izumi Naka, Jun Ohashi, Jintana Patarapotikul

There are errors in the alignment of the data presented in Tables 2–6. Please see the corrected Tables 2–6 within S1 File below.

## Supporting information

S1 File Corrected versions of Tables 2–6. (PDF)
