# Supplementary material for: Sequence variation in Plasmodium falciparum merozoite surface protein-2 is associated with virulence causing severe and cerebral malaria
Source: PLoS One. 2018 Jan 17;13(1):e0190418. doi: 10.1371/journal.pone.0190418 (PMC5771562; doi:10.1371/journal.pone.0190418)
Supplement: S1 Fig — Sequence analysis differentiated msp2 variants into FC27 (red bars) or 3D7 (blue bars) families, respectively. The frequencies of variants sized by gel electrophoresis is shown by green bars in the lower panel. (PDF) [file pone.0190418.s001.pdf]

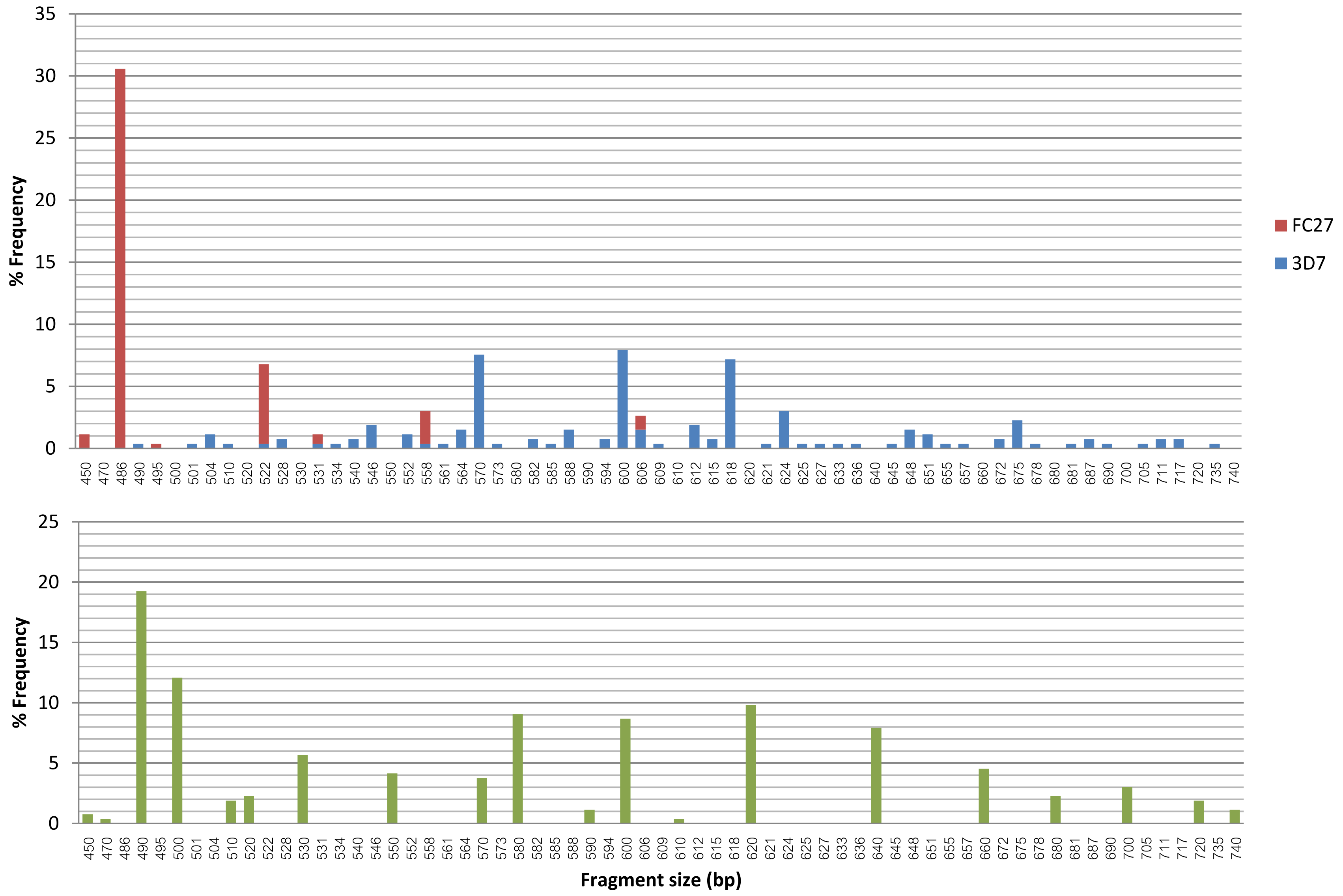

S1 Fig. Frequencies of different sized *msp2* variants determined by DNA sequencing (upper) and conventional gel electrophoresis (lower). Sequence analysis differentiated *msp2* variants into FC27 (red bars) or 3D7 (blue bars) families, respectively. The frequencies of variants sized by gel electrophoresis is shown by green bars in the lower panel.
