# Supplementary material for: Sequence variation in Plasmodium falciparum merozoite surface protein-2 is associated with virulence causing severe and cerebral malaria
Source: PLoS One. 2018 Jan 17;13(1):e0190418. doi: 10.1371/journal.pone.0190418 (PMC5771562; doi:10.1371/journal.pone.0190418)
Supplement: S1 Table — (PDF) [file pone.0190418.s002.pdf]

S1 Table. **Genetic diversity of R1 region found in 163 3D7-liked *msp2* sequences**.

Code	Dipeptide
1	GA
2	GS
3	GG
4	GD
5	SA
6	VA
7	RD
8	SG
9	GT
0	PT

Allele	R1 sequence	R1 type
1	11621116211165	162
2	116211162111621165	162
3	1162116211621111162115	162
4	16211162111621111162111621165	162
5	11621162116211111621116621165	162
6	162111621116211111621111621165	162
7	116211621621116211116211162115	162
8	1162116211621111621116211162115	162
9	11621162116211116211116211162115	162
10	116211621162111116211116211162115	162
11	18511851185 21	185
12	1851185118518521	185
13	18511851185185185185185185185 21	185
14	185118511851851851851851851851 8521	185
15	1851118518518518518518518518518521	185
16	1851111851185118511851851185851 8521	185
17	185118511851851851851851851851851 8521	185
18	185118511858511851185118511851185118511 8521	185
19	18511851135185118511851185118511851185118521	185
20	1851185118518511185118511851185118511851118521	185
21	185118511851185118511851185118511851185185118511 8521	185
22	18511851185118511851118511851185118518511851185118521	185
23	185118511851185118511185118511851185185185118511 [illegible]	185
24	01118524185851858518585 1	18585
25	011118524185851858518585185851	18585
26	011118524185851858518585185851 85185851	18585
27	0011111852418585185851858518585185851	18585
28	011111852418585185851858518585185851	18585
29	185111858518585185851858518511 18521	18585
30	918521185241 65	1852
31	9185211852118524165	1852
32	18521652165	2165
33	165216521652165	2165
34	185216521652165	2165
35	185216521652165 21652165	2165
36	18521652165216521652165 21652165	2165
37	1852165216521652165216521652165 21652165	2165
38	11185271652716527165271 65	27165
39	111852716527165271652716527165271 65	27165
40	1118527165271652716527165271652716527165	27165
41	11185271652716527165271652716527165271652716527165	27165
42	138538538524	385
43	138538538538538538524	385
44	138538538538538538521	385
45	138538538538538538538524	385
46	138538538538538538538538524	385
47	138538538538538538538538538 5	385
48	138538538538538538538538538538524	385
49	1385385385385385385385385385385385385385 24	385
50	13851385138513851385138513851385185118511 8521	385
51	18535353535353535353535353535353535353535 24	35
52	1353535353535353535353535353535353535353524	35
53	18535353535353535353535353535353535353535353535353524	35
54	1385385385385 35353524	385-35
