# Supplementary material for: Correction: Sequence variation in Plasmodium falciparum merozoite surface protein-2 is associated with virulence causing severe and cerebral malaria
Source: PLoS One. 2018 Apr 25;13(4):e0196694. doi: 10.1371/journal.pone.0196694 (PMC5919017; doi:10.1371/journal.pone.0196694)
Supplement: S1 File — (PDF) [file pone.0196694.s001.pdf]

Table 2. Frequencies of FC27 and 3D7-like *msp2* in *P. falciparum* isolates from 277 malaria patients in Thailand with mild (M), severe (S) or cerebral (C) disease.

***Msp2* family**	**Mild (M) N=115 (%)**	**Severe (S) N=84 (%)**	**Cerebral (C) N=78 (%)**	**Total N=277 (%)**	**M vs S *P*-value Odds Ratio**	**M vs C *P*-value Odds Ratio**	**S vs C *P*-value Odds Ratio**
FC27	54 (47.0)	28 (33.3)	32 (41.0)	114 (41.2)	*P* = 0.054	*P* = 0.416	*P* = 0.311
3D7	61 (53.0)	56 (66.7)	46 (59.0)	163 (58.8)	OR = 0.56	OR = 0.79	OR = 1.39

Table 3. Allele frequencies of polymorphisms in FC27-like *msp2* of *P. falciparum* isolates from mild, severe and cerebral malaria patients in Thailand.

Region	Polymorphic position[a]		Mild	Severe	Cerebral	Total	M vs S[d]	M vs C	S vs C
	Nucleotide	Codon (aa.)	(%)	(%)	(%)	(%)	*P*-value, OR	*P*-value,OR	*P*-value,OR
Block 2	23 A/C[b]	8 AAG (K)	19 (35.2)	7 (25.0)	13 (40.6)	39 (34.2)	0.347, 0.61	0.614, 1.26	0.200, 2.05
	**24 G/T**	**· · T (N)**	**13 (24.1)**	**13 (46.4)**	**7 (21.9)**	**33 (28.9)**	**0.039, 2.73**	**0.816, 0.88**	**0.044, 0.32**
		· CT (T)	22 (40.7)	8 (28.6)	12 (37.5)	42 (36.8)	0.278, 0.58	0.766, 0.87	0.464, 1.50
	27 T/G	9 AGT (S)	45 (83.3)	18 (64.3)	26 (81.3)	89 (78.1)	0.053, 2.78	0.806, 1.15	0.138, 0.42
		· ·G (R)	9 (16.7)	10 (35.7)	6 (18.8)	25 (21.9)			
	32 G/A	11 GGT (G)	51 (94.4)	25 (89.3)	31 (96.9)	107 (93.9)	NA.	NA.	NA.
		· A · (D)	3 (5.6)	3 (10.7)	1 (3.1)	7 (6.1)			
	37 A/G	13 AAT (N)	50 (92.6)	25 (89.3)	31 (96.9)	106 (93.0)	NA.	NA.	NA.
	39 T/A	· · A (K)	3 (5.6)	3 (10.7)	1 (3.1)	7 (6.1)	NA.	NA.	NA.
		G · · (D)	1 (1.9)	0 (0)	0 (0)	1 (0.9)	NA.	NA.	NA.
	48 A/T	16 AAA (K)	33 (61.1)	18 (64.3)	21 (65.6)	72 (63.2)	0.779, 0.87	0.676, 0.82	0.914, 0.94
		· · T (N)	21 (38.9)	10 (35.7)	11 (34.4)	42 (36.8)			
	49_57indel	17_19 del	53 (98.1)	26 (92.9)	32 (100.0)	111 (97.4)	NA.	NA.	NA.
		17_19 ins GCT CCA AAA (APK)	0 (0)	2 (7.1)	0 (0)	2 (1.8)	NA.	NA.	NA.
		17_19 ins GCT CCA AAT (APN)	1 (1.9)	0 (0)	0 (0)	1 (0.9)	NA.	NA.	NA.
Block 3[c]	**(R1)$_n$**(R2)$_n$	***(R1)***(R2)(R2)	1 (1.9)	2 (7.1)	0 (0)	3 (2.6)	NA.	NA.	NA.
		(R1)(R2)(R2)(R2)	39 (72.2)	22 (78.6)	21 (65.6)	82 (71.9)	0.532, 1.41	0.520, 0.73	0.267, 0.52
		(R1)(R2)(R2)(R2)(R2)	7 (13.0)	3 (10.7)	9 (28.1)	19 (16.7)	0.768, 0.81	0.081, 2.63	0.093, 3.26
		(R1)(R2)(R2)(R2)(R2)(R2)	5 (9.3)	1 (3.6)	1 (3.1)	7 (6.1)	NA.	NA.	NA.
		(R1)(R1)(R1)(R2)	2 (3.7)	0 (0)	1 (3.1)	3 (2.6)	NA.	NA.	NA.
	R1xR2	A 12	1 (1.9)	0 (0)	0 (0)	1 (0.9)	NA.	NA.	NA.
		A 122	20 (37.0)	15 (53.6)	11 (34.4)	46 (40.3)	0.151, 1.96	0.804, 0.89	0.134, 0.45
		**A 1222**	**6 (11.1)**	**0 (0)**	**7 (21.9)**	**13 (11.4)**	**0.066**	**0.178, 2.24**	**0.009**
		A 132	2 (3.7)	1 (3.6)	0 (0)	3 (2.6)	NA.	NA.	NA.
		A 1333	0 (0)	2 (7.1)	0 (0)	2 (1.8)	NA.	NA.	NA.
		A 222	3 (5.6)	0 (0)	0 (0)	3 (2.6)	NA.	NA.	NA.
		A 333	14 (25.9)	6 (21.4)	10 (31.3)	30 (26.3)	0.653, 0.78	0.595, 1.30	0.391, 1.67
		A 3333	0 (0)	0 (0)	2 (6.3)	2 (1.8)	NA.	NA.	NA.
		A 33333	5 (9.3)	1 (3.6)	1 (3.1)	7 (6.1)	NA.	NA.	NA.
		B 22	0 (0)	2 (7.1)	0 (0)	2 (1.8)	NA.	NA.	NA.
		B 2222	0 (0)	1 (3.6)	0 (0)	1 (0.9)	NA.	NA.	NA.
		C 2111	1 (1.9)	0 (0)	0 (0)	1 (0.9)	NA.	NA.	NA.
		ADD 2	2 (3.7)	0 (0)	1 (3.1)	3 (2.6)	NA.	NA.	NA.
Block 4	58 G/A	20 GAA (E)	43 (79.6)	24 (85.7)	30 (93.8)	97 (85.1)	0.499, 0.65	0.077, 0.26	0.301, 0.4
		A · · (K)	11 (20.4)	4 (14.3)	2 (6.3)	17 (14.9)			
	64 C/A	22 CAA (Q)	44 (81.5)	24 (85.7)	30 (93.8)	98 (86.0)	0.629, 0.73	0.113, 0.29	0.301, 0.4
		A · · (K)	10 (18.5)	4 (14.3)	2 (6.3)	16 (14.0)			

[a] Position relative to the first nucleotide / aa. of each block (Fig 2).

[b] In case of SNPs, alleles found in the *msp2* sequence of K1 (FC27-liked) (M59766.1) / another found in our data set was shown and amino acid (aa.) changes were indicated.

[c] Variation in number of repeat 1 and 2 [$(R1)_n(R2)_n$] generated 5 distinct alleles in block 3, while 13 alleles were detected when sequence variation in repeat units were considered [R1xR2].

[d] Allele frequencies were compared between mild (M) and severe (S), mild and cerebral (C), as well as severe and cerebral. For bi-allelic polymorphisms, the odds ratio (OR) of a minor-frequency allele for risk to severe and cerebral malaria by comparing to a major allele was analyzed. For polymorphisms with more than 2 alleles, the presence or absence of individual alleles were compared. OR and *P*-values are shown, with significant values in red bold. NA. (not applicable) indicates bi-allelic polymorphisms with minor allele frequency <10% and individual alleles having frequencies <10% or >90%, in which their associations with malaria severity were not analyzed. OR was undefined in cases of zero cell count.

Table 4. Haplotype frequencies of *P. falciparum* FC27-like *msp2* of from mild, severe and cerebral malaria patients in Thailand, comprising polymorphisms in block 2, 3 and 4.

FC27 haplotype	Amino acid changes[a]	Mild (%)	Severe (%)	Cerebral (%)	Total (%)	M vs S[c] *P*-value, OR	M vs C *P*-value, OR	S vs C *P*-value, OR
Bl.2 - Bl.3 - Bl.4	8 - 9 -11 -13 -16 -indel - R1R2 - 20 - 22							
Haplotype 1[b]	K S G N K del A333 E Q	14 (25.9)	6 (21.4)	10 (31.3)	30 (26.3)	0.653, 0.78	0.595, 1.30	0.39, 1.67
2	· · · · · del A3333 · ·	0 (0)	0 (0)	2 (6.3)	2 (1.8)	NA.	NA.	NA.
3	· · · · · del A33333 · ·	5 (9.3)	1 (3.6)	1 (3.1)	7 (6.1)	NA.	NA.	NA.
4	T · · · · del A122 · ·	3 (5.6)	0 (0)	2 (6.3)	5 (4.4)	NA.	NA.	NA.
5	T · · · N del A122 · ·	1 (1.9)	3 (10.7)	1 (3.1)	5 (4.4)	NA.	NA.	NA.
**6[b]**	**T · · · N del A1222 · ·**	**5 (9.3)**	**0 (0)**	**7 (21.9)**	**12 (10.5)**	**0.097**	**0.103, 2.75**	**0.009**
7	T · · · N del A132 · ·	2 (3.7)	1 (3.6)	0 (0)	3 (2.6)	NA.	NA.	NA.
8	T · · · N del A1222 K ·	1 (1.9)	0 (0)	0 (0)	1 (0.9)	NA.	NA.	NA.
9	T · · · N del A12 K K	1 (1.9)	0 (0)	0 (0)	1 (0.9)	NA.	NA.	NA.
10[b]	T · · · N del A122 K K	8 (14.8)	4 (14.3)	2 (6.3)	14 (12.3)	0.949, 0.96	0.231, 0.38	0.301, 0.4
11	T R · · N del A122 · ·	1 (1.9)	0 (0)	0 (0)	1 (0.9)	NA.	NA.	NA.
12	N · · · N APN A222 K K	1 (1.9)	0 (0)	0 (0)	1 (0.9)	NA.	NA.	NA.
13	N · · D · del C2111 · ·	1 (1.9)	0 (0)	0 (0)	1 (0.9)	NA.	NA.	NA.
14	N · D K · del ADD2 · ·	1 (1.9)	0 (0)	0 (0)	1 (0.9)	NA.	NA.	NA.
15	N · D K · del B22 · ·	0 (0)	2 (7.1)	0 (0)	2 (1.8)	NA.	NA.	NA.
16	N · D K · del A222 · ·	1 (1.9)	0 (0)	0 (0)	1 (0.9)	NA.	NA.	NA.
17	N · D K · del B2222 · ·	0 (0)	1 (3.6)	0 (0)	1 (0.9)	NA.	NA.	NA.
18	N · D K N del ADD2 · ·	1 (1.9)	0 (0)	1 (3.1)	2 (1.8)	NA.	NA.	NA.
19[b]	N R · · · del A122 · ·	7 (13.0)	8 (28.6)	6 (18.8)	21 (18.4)	0.083, 2.69	0.469, 1.55	0.370, 0.58
20	N R · · · del A222 · ·	1 (1.9)	0 (0)	0 (0)	1 (0.9)	NA.	NA.	NA.
21	N R · · N APK A1333 · ·	0 (0)	2 (7.1)	0 (0)	2 (1.8)	NA	NA.	NA.

[a] Position relative to the first aa. of each block (Fig 2). Blocks are shaded using different colors.

[b] Major haplotypes (frequency ≥ 10%) observed in the parasite population that were analyzed for association with malaria severity.

[c] Haplotype frequencies were compared between mild (M) and severe (S), mild and cerebral (C), and severe and cerebral. *P*-value and Odds ratios (OR) are shown, with statistically significant differences in red bold. NA. (not applicable) indicates haplotypes with frequencies >10% whose association with malaria severity was not analyzed. OR was undefined in cases of zero cell count.

Table 5. Allele frequencies of polymorphisms in the 3D7-like *msp2* sequences of *P. falciparum* isolates from mild, severe and cerebral malaria patients in Thailand.

Region	Polymorphic position[a]		Mild	Severe	Cerebral	Total	M vs S[e]	M vs C	S vs C
	**Nucleotide**	**Codon (aa.)**	**(%)**	**(%)**	**(%)**	**(%)**	***P*-value, OR**	***P*-value, OR**	***P*-value, OR**
Block 2	12 G/T[b]	4 AAG (K)<br>· · T (N)	26 (43.3)<br>34 (56.7)	24 (45.3)<br>29 (54.7)	16 (37.2)<br>27 (62.8)	66 (42.3)<br>90 (57.7)	0.835, 1.08	0.533, 0.77	0.425, 0.72
	13 C/A<br>14 C/T	5 CCT (P)<br>A · · (T)<br>· T · (L)	48 (78.7)<br>11 (18.0)<br>2 (3.3)	47 (83.9)<br>8 (14.3)<br>1 (1.8)	36 (83.7)<br>7 (16.3)<br>0 (0)	131 (81.9)<br>26 (16.3)<br>3 (1.9)	0.469, 1.41<br>0.583, 0.76<br>NA.	0.521, 1.39<br>0.816, 0.88<br>NA.	0.978, 0.98<br>0.784, 1.17<br>NA.
	16 T/C	6 TCT (S)<br>C · · (P)	7 (11.5)<br>54 (88.5)	7 (12.5)<br>49 (87.5)	7 (16.3)<br>36 (83.7)	21 (13.1)<br>139 (86.9)	0.865, 1.10	0.480, 1.50	0.593, 1.36
Block 3	**R1 region[c]**	162 : GAVAGS<br>185 : GASGSA<br>1852 : GASGSAGS<br>18585 : GASGSASGSA<br>2165 : GSGAVASA<br>27165 : GSRDGAVASA<br>35 : GGSA<br>385 : GGSGSA<br>385_35: GGSGSA GGSA	14 (23.0)<br>10 (16.4)<br>7 (11.5)<br>6 (9.8)<br>6 (9.8)<br>6 (9.8)<br>5 (8.2)<br>6 (9.8)<br>1 (1.6)	11 (19.6)<br>8 (14.3)<br>13 (23.2)<br>6 (10.7)<br>3 (5.4)<br>6 (10.7)<br>2 (3.6)<br>7 (12.5)<br>0 (0)	11 (23.9)<br>8 (14.3)<br>5 (10.9)<br>6 (13.0)<br>2 (4.3)<br>2 (4.3)<br>4 (8.7)<br>8 (17.4)<br>0 (0)	36 (22.1)<br>26 (16.0)<br>25 (15.3)<br>18 (11.0)<br>11 (6.7)<br>14 (8.6)<br>11 (6.7)<br>21 (12.9)<br>1 (0.6)	0.663, 0.82<br>0.752, 0.85<br>0.092, 2.33<br>0.876, 1.11<br>NA.<br>NA.<br>NA.<br>0.647, 1.31<br>NA.	0.907, 1.06<br>0.891, 1.07<br>0.922, 0.94<br>0.603, 1.38<br>NA.<br>NA.<br>NA.<br>0.251, 1.93<br>NA.	0.602, 1.29<br>0.668, 1.26<br>0.104, 0.40<br>0.716, 1.25<br>NA.<br>NA.<br>NA.<br>0.488, 1.47<br>NA.
	**NR region**<br>1_30 indel	 1_10 ins GNGANPGADA<br>**1_10 ins RNGANPGADA**<br>3_6 del GN - - - - GADA<br>1_8 del - - - - - - - - - DA<br>1_10 del - - - - - - - - - -	 18 (29.5)<br>**13 (21.3)**<br>14 (23.0)<br>9 (14.8)<br>7 (11.5)	 12 (21.4)<br>**19 (33.9)**<br>11 (19.6)<br>6 (10.7)<br>8 (14.3)	 13 (28.3)<br>**7 (15.2)**<br>11 (23.9)<br>9 (19.6)<br>6 (13.0)	 43 (26.4)<br>**39 (23.9)**<br>36 (22.1)<br>24 (14.7)<br>21 (12.9)	 0.317, 0.65<br>**0.126, 1.90**<br>0.663, 0.82<br>0.514, 0.69<br>0.650, 1.29	 0.888, 0.94<br>**0.423, 0.66**<br>0.907, 1.06<br>0.510, 1.41<br>0.806, 1.16	 0.425, 1.44<br>**0.031, 0.35**<br>0.602, 1.29<br>0.209, 2.03<br>0.856, 0.90
	31 G/A	11 GAG (E)<br>A · · (K)	46 (75.4)<br>15 (24.6)	44 (78.6)<br>12 (21.4)	33 (71.7)<br>13 (28.3)	23 (75.5)<br>40 (24.5)	0.685, 0.84	0.669, 1.21	0.425, 1.44
	34 A/G	12 AGA (R)<br>G · · (G)	35 (57.4)<br>26 (42.6)	30 (53.6)<br>26 (46.4)	31 (67.4)<br>15 (32.6)	96 (58.9)<br>67 (41.1)	0.679, 1.17	0.292, 0.65	0.157, 0.56
	40 C/T	14 CCA (P)<br>**T · · (S)**	41 (67.2)<br>**20 (32.8)**	35 (62.5)<br>**21 (37.5)**	37 (80.4)<br>**9 (19.6)**	113 (69.3)<br>**50 (30.7)**	**0.594, 1.23**	**0.128, 0.50**	**0.048, 0.41**
	50 C/G	17 CCC (P)<br>· G · (R)	60 (98.4)<br>1 (1.6)	56 (100.0)<br>0 (0)	44 (95.7)<br>2 (4.3)	160 (98.2)<br>3 (1.8)	NA.	NA.	NA.
	52 G/A	18 GCT (A)<br>A · · (T)	61 (100)<br>0 (0)	56 (100)<br>0 (0)	45 (97.8)<br>1 (2.2)	162 (99.4)<br>1 (0.6)	NA.	NA.	NA.
	**R2 region[d]**<br>(ACT ACC ACA)$_2$<br>(ACT ACC ACA)$_3$<br>**(ACT ACC ACA)$_4$**	 (T)$_8$<br>(T)$_{11}$<br>**(T)$_{14}$**	 42 (68.9)<br>8 (13.1)<br>**11 (18.0)**	 37 (66.1)<br>4 (7.1)<br>**15 (26.8)**	 35 (76.1)<br>7 (15.2)<br>**4 (8.7)**	 114 (69.9)<br>19 (11.7)<br>**30 (18.7)**	 0.748, 0.88<br>0.288, 0.51<br>**0.255, 1.66**	 0.410, 1.44<br>0.757, 1.19<br>**0.168, 0.43**	 0.269, 1.63<br>0.191, 1.33<br>**0.020, 0.26**

Table 5. (continued)

Region	Polymorphic position[a]		Mild	Severe	Cerebral	Total	M vs S[e]	M vs C	S vs C
	Nucleotide	Codon (aa.)	(%)	(%)	(%)	(%)	*P*-value, OR	*P*-value, OR	*P*-value, OR
Block 4	40 C/T	14 CCA (P)<br>T · · (S)	55 (90.2)<br>6 (9.8)	50 (89.3)<br>6 (10.7)	44 (95.7)<br>2 (4.3)	149 (91.4)<br>14 (8.6)	NA.	NA.	NA.
	**51 A/T**	**17 AAA (K)**<br>· · T (N)	**5 (8.2)**<br>56 (91.8)	**7 (12.5)**<br>49 (87.5)	**11 (23.9)**<br>35 (76.1)	**23 (14.1)**<br>140 (85.9)	**0.443, 1.60**	**0.024, 3.52**	**0.132, 2.20**
	58 G/A	20 GAA (E)<br>A · · (K)	38 (62.3)<br>23 (37.7)	39 (69.6)<br>17 (30.4)	31 (67.4)<br>15 (32.6)	108 (66.3)<br>55 (33.7)	0.403, 0.72	0.586, 0.80	0.807, 1.11
	78 A/T	26 AAA (K)<br>· · T (N)	30 (53.6)<br>26 (46.4)	35 (64.8)<br>19 (35.2)	28 (68.3)<br>13 (31.7)	93 (61.6)<br>58 (38.4)	0.231, 0.62	0.144, 0.54	0.722, 0.86
	82 G/C/A<br>83 A/G	28 GAA (E)<br>· G · (G)<br>C · · (Q)<br>A · · (K)	29 (51.8)<br>17 (30.4)<br>7 (12.5)<br>3 (5.4)	34 (63.0)<br>12 (22.2)<br>4 (7.4)<br>4 (7.4)	27 (65.9)<br>10 (24.4)<br>2 (4.9)<br>2 (4.9)	90 (59.6)<br>39 (25.8)<br>13 (8.6)<br>9 (6.0)	0.236, 1.58<br>0.333, 0.66<br>NA.<br>NA.	0.166, 1.80<br>0.517, 0.74<br>NA.<br>NA.	0.771, 1.13<br>0.804, 1.13<br>NA.<br>NA.
	91_93 indel	31 ins GAA (E)<br>ins AAA (K)<br>del	26 (46.4)<br>24 (42.9)<br>6 (10.7)	27 (50.0)<br>24 (44.4)<br>3 (5.6)	15 (36.6)<br>25 (61.0)<br>1 (2.4)	68 (45.0)<br>73 (48.3)<br>10 (6.6)	0.867, 1.07<br>0.837, 1.08<br>NA.	0.078, 2.08<br>0.332, 0.67<br>NA.	0.110, 1.95<br>0.242, 0.62<br>NA.
	95 C/A	32 CCA (P)<br>· A · (Q)	51 (91.1)<br>5 (8.9)	43 (79.6)<br>11 (20.4)	37 (90.2)<br>4 (9.8)	131 (86.8)<br>20 (13.2)	0.089, 2.61	0.890, 1.10	0.160, 0.42
	67_99 indel	23_33 insert<br>23_33 deletion	56 (91.8)<br>5 (8.2)	54 (96.4)<br>2 (3.6)	41 (89.1)<br>5 (10.9)	151 (92.6)<br>12 (7.4)	NA.	NA.	NA.

[a] Position relative to the first nucleotide / aa. of each block (Fig 3)

[b] In case of SNPs, alleles found in the *msp2* sequence of 3D7 (PFB0300c) / another allele found in our data set was shown, and amino acid (aa.) changes are indicated.

[c] For The R1 region in block 3, sequences can be grouped into nine types according to the presence of different types of numerically coded dipeptide motifs (Table S1).

[d] For the R2 region, there were 8, 11, and 14 Threonine repeats encoded by 2-4 copies of nanomer (ACT ACC ACA) followed by ACT ACT.

[e] Allele frequencies were compared between mild (M) and severe (S), mild and cerebral (C), and severe and cerebral. For bi-allelic polymorphisms, the odds ratios (OR) of minor-frequency alleles compared to major alleles associated with severe and cerebral malaria were analyzed. For

polymorphisms with more than 2 alleles, the presence/absence of individual alleles were compared. OR and *P*-values are shown, with significant differences in red bold**.** NA. (not applicable) indicates bi-allelic polymorphisms with a minor allele frequency <10% and individual alleles with frequencies <10% or >90%, in which their association with malaria severity were not analyzed**.**

Table 6. Haplotype frequencies of 3D7 like *msp2* of *P. falciparum* from mild, severe and cerebral malaria patients in Thailand, with each block analyzed separately**.**

3D7 haplotype	Amino acid changes[a]	Mild (%)	Severe (%)	Cerebral (%)	Total (%)	M vs S[c] *P*-value, OR	M vs C *P*-value, OR	S vs C *P*-value, OR
**Block 2**	4 – 5 – 6							
Haplotype 1[b]	N P P	27 (45.0)	22 (41.5)	20 (46.5)	69 (44.2)	0.709, 0.87	0.879, 1.06	0.623, 1.23
2[b]	· · S	7 (11.7)	7 (13.2)	7 (16.3)	21 (13.5)	0.804, 1.15	0.501, 1.47	0.672, 1.28
3[b]	K · ·	13 (21.7)	17 (32.1)	9 (20.9)	39 (25.0)	0.211, 1.71	0.928, 0.96	0.222, 0.56
4[b]	K T ·	11 (18.3)	6 (11.3)	7 (16.3)	24 (15.4)	0.298, 0.57	0.787, 0.87	0.480, 1.52
5	K L ·	2 (3.3)	1 (1.9)	0 (0)	3 (1.9)	NA.	NA.	NA.
**Block 3** NR-R2	1_10indel -11 - 12 - 14 - 17 - 18 - [T]							
Haplotype 1[b]	Ins G E R P P A 8	11 (18.0)	8 (14.3)	10 (21.7)	29 (17.8)	0.583, 0.76	0.633, 1.26	0.326, 1.67
2	Ins G · G S · · 14	4 (6.6)	3 (5.4)	0 (0)	7 (4.3)	NA.	NA.	NA.
3	Ins G · G S · · 11	1 (1.6)	0 (0)	0 (0)	1 (0.6)	NA.	NA.	NA.
4	Ins G · G S R · 11	1 (1.6)	0 (0)	2 (4.3)	3 (1.8)	NA.	NA.	NA.
5	Ins G K · · · · 8	1 (1.6)	1 (1.8)	1 (2.2)	3 (1.8)	NA.	NA.	NA.
6	Ins R · · · · · 11	0 (0)	1 (1.8)	0 (0)	1 (0.6)	NA.	NA.	NA.
7	Ins R · G S · · 8	6 (9.8)	6 (10.7)	2 (4.3)	14 (8.6)	NA.	NA.	NA.
8[b]	Ins R · G S · · 14	6 (9.8)	12 (21.4)	4 (8.7)	22 (13.5)	0.083, 2.50	0.841, 0 .87	0.079, 0.35
9	Ins R · G S · · 11	1 (1.6)	0 (0)	1 (2.2)	2 (1.2)	NA.	NA.	NA.
10[b]	Del3_6 K · · · · 8	14 (23.0)	11 (19.6)	11 (23.9)	36 (22.1)	0.663, 0.82	0.907, 1.06	0.602, 1.29
11	Del1_8 · · · · · 8	4 (6.6)	6 (10.7)	4 (8.7)	14 (8.6)	NA.	NA.	NA.
12	Del1_8 · · · · · 11	5 (8.2)	3 (5.4)	4 (8.7)	12 (7.4)	NA.	NA.	NA.
13	Del1_8 · G S · · 14	1 (1.6)	0 (0)	0 (0)	1 (0.6)	NA.	NA.	NA.
14	Del1_8 K · · · T 8	0 (0)	0 (0)	1 (2.2)	1 (0.6)	NA.	NA.	NA.
15[b]	Del1_10 · G · · · 8	6 (9.8)	5 (8.9)	6 (13.0)	17 (10.4)	0.867, 0.90	0.603,1.38	0.505, 1.53
**Block 4**	14 – 17 – 20 – 26 – 28 – 31 – 32							
Haplotype 1[b]	P N E K E K P	7 (11.5)	8 (14.3)	6 (13.0)	21 (12.9)	0.650, 1.29	0.806, 1.16	0.856, 0.90
2[b]	· · · · · E Q	5 (8.2)	11 (19.6)	4 (8.7)	20 (12.3)	0.072, 2.74	0.927, 1.07	0.120, 0.39
3	· · · · Q E ·	7 (11.5)	4 (7.1)	2 (4.3)	13 (8.0)	NA.	NA.	NA.
4	· · · N K · ·	1 (1.6)	0 (0)	0 (0)	1 (0.6)	NA.	NA.	NA.
5	· · · N K E ·	2 (3.3)	1 (1.8)	1 (2.2)	4 (2.5)	NA.	NA.	NA.
6	· · K · · · ·	6 (9.8)	5 (8.9)	5 (10.9)	16 (9.8)	NA.	NA.	NA.
7	· · K N G · ·	5 (8.2)	4 (7.1)	3 (6.5)	12 (7.4)	NA.	NA.	NA.
8[b]	· · K N G E ·	12 (19.7)	8 (14.3)	7 (15.2)	27 (16.6)	0.439, 0.68	0.551, 0.73	0.895, 1.08
**9[b]**	**· K · · · · ·**	**5 (8.2)**	**7 (12.5)**	**11 (23.9)**	**23 (14.1)**	**0.443, 1.60**	**0.024, 3.52**	**0.132, 2.20**
10	S · · N · del ·	6 (9.8)	3 (5.4)	1 (2.2)	10 (6.1)	NA.	NA.	NA.
11	S · · N K · ·	0 (0)	3 (5.4)	1 (2.2)	4 (2.5)	NA.	NA.	NA.
12	· · · ---- 23_33 del ----	5 (8.2)	2 (3.6)	5 (10.9)	12 (7.4)	NA.	NA.	NA.

[a] Position relative to the first aa. of each block (Fig 3).

[b] Major haplotypes (frequency ≥ 10%) observed in the parasite population that were analyzed for association with malaria severity.

[c] Haplotype frequencies were compared between mild (M) and severe (S), mild and cerebral (C), as well as severe and cerebral. *P*-values and odds ratios (OR) are shown, with significant differences in red bold. NA. (not applicable) indicates haplotypes with frequencies >10% whose associations with malaria severity were not analyzed.
